# Divergent epigenetic responses to perinatal asphyxia in severe mental disorders

**DOI:** 10.1038/s41398-023-02709-7

**Published:** 2024-01-08

**Authors:** Laura A. Wortinger, Anne-Kristin Stavrum, Alexey A. Shadrin, Attila Szabo, Sondre Høeg Rukke, Stener Nerland, Runar Elle Smelror, Kjetil Nordbø Jørgensen, Claudia Barth, Dimitrios Andreou, Melissa A. Weibell, Srdjan Djurovic, Ole A. Andreassen, Marianne Thoresen, Gianluca Ursini, Ingrid Agartz, Stephanie Le Hellard

**Affiliations:** 1 Department of Psychiatric Research, Diakonhjemmet Hospital, Oslo, Norway; 2 NORMENT, Institute of Clinical Medicine, University of Oslo, Oslo, Norway; 3 NORMENT, Department of Clinical Science, University of Bergen, Bergen, Norway; 4 Dr. Einar Martens Research Group for Biological Psychiatry, Center for Medical Genetics and Molecular Medicine, Haukeland University Hospital, Bergen, Norway; 5 NORMENT, Division of Mental Health and Addiction, Oslo University Hospital, Oslo, Norway; 6 KG Jebsen Centre for Neurodevelopmental Disorders, University of Oslo, Oslo, Norway; 7 Faculty of Medicine, University of Bergen, Bergen, Norway; 8 Department of Psychiatry, Telemark Hospital, Skien, Norway; 9 Centre for Psychiatry Research, Department of Clinical Neuroscience, Karolinska Institutet and Stockholm Health Care Services, Stockholm County Council, Stockholm, Sweden; 10 TIPS—Network for Clinical Research in Psychosis, Department of Psychiatry, Stavanger University Hospital, Stavanger, Norway; 11 Faculty of Health, Network for Medical Sciences, University of Stavanger, Stavanger, Norway; 12 Department of Medical Genetics, Oslo University Hospital, Oslo, Norway; 13 Department of Physiology, Institute of Basic Medical Sciences, University of Oslo, Oslo, Norway; 14 Neonatal Neuroscience, Translational Health Sciences, University of Bristol, Bristol, United Kingdom; 15 Lieber Institute for Brain Development, Johns Hopkins Medical Campus, Baltimore, MD USA; 16 Department of Psychiatry and Behavioral Sciences, Johns Hopkins University School of Medicine, Baltimore, MD USA

**Keywords:** Genetics, Neuroscience

## Abstract

Epigenetic modifications influenced by environmental exposures are molecular sources of phenotypic heterogeneity found in schizophrenia and bipolar disorder and may contribute to shared etiopathogenetic mechanisms of these two disorders. Newborns who experienced perinatal asphyxia have suffered reduced oxygen delivery to the brain around the time of birth, which increases the risk of later psychiatric diagnosis. This study aimed to investigate DNA methylation in blood cells for associations with a history of perinatal asphyxia, a neurologically harmful condition occurring within the biological environment of birth. We utilized prospective data from the Medical Birth Registry of Norway to identify incidents of perinatal asphyxia in 643 individuals with schizophrenia or bipolar disorder and 676 healthy controls. We performed an epigenome wide association study to distinguish differentially methylated positions associated with perinatal asphyxia. We found an interaction between methylation and exposure to perinatal asphyxia on case–control status, wherein having a history of perinatal asphyxia was associated with an increase of methylation in healthy controls and a decrease of methylation in patients on 4 regions of DNA important for brain development and function. The differentially methylated regions were observed in genes involved in oligodendrocyte survival and axonal myelination and functional recovery (LINGO3); assembly, maturation and maintenance of the brain (BLCAP;NNAT and NANOS2) and axonal transport processes and neural plasticity (SLC2A14). These findings are consistent with the notion that an opposite epigenetic response to perinatal asphyxia, in patients compared with controls, may contribute to molecular mechanisms of risk for schizophrenia and bipolar disorder.

## Introduction

Schizophrenia (SZ) and bipolar disorders (BD) are highly heritable (60-85%) [1–3] and are often regarded as part of a clinical continuum [4]. Some of the heritability may be attributed to an overlapping polygenic architecture [5–8] between the disorders and to epigenetic modifications [9–12] influenced by environmental exposures throughout development, starting as early as the prenatal period [13, 14]. Adverse events during pregnancy and birth are some of the best-replicated environmental risk factors for psychosis [13], and of these adverse events, fetal hypoxia is among those most consistently implicated [15–17]. Perinatal asphyxia (PA) is a condition where affected newborns experience a deprivation of oxygen (i.e., hypoxia) in the whole body including the brain, with or without concomitance of reduced cerebral blood flow. Population-based studies have found that a history of PA increases the risk of developing SZ (Odds Ratio = 4.4) [13, 18] and BD (Hazard Ratio = 5.3) [19], consistent with evidence of an altered response to hypoxia and oxidative stress that influenced brain development [20–22]. Most individuals with a history of PA do not develop any psychiatric condition. However, factors underlying the effect of PA on a trajectory of resilience or on a trajectory of altered neurodevelopment linked with psychiatric disorders are still unknown.

Hypoxia modulates a cascade of systemic and cell/tissue specific hormonal and molecular homeostatic responses in the body. Having experienced perinatal hypoxia impacts developmental plasticity (i.e., the ability of genes to be differentially expressed according to environmental cues) and has been linked to increased risk of disease later in life such as diabetes, inflammation, cardiac dysfunction, hypertension, atherosclerosis, accelerated age-related cognitive decline and other neurological conditions [23]. This indicates that PA can induce complex broad system-level responses, which could be revealed by changes at the level of peripheral tissues, such as epigenetic modifications.

Epigenetic modifications, such as DNA methylation (DNAm), influence gene expression by the recruitment of methyl-binding proteins and disruption of transcription factor binding that initiate gene silencing and chromatin compaction [24]. Both increases and decreases in gene expression are associated with DNAm [25], which has initiated a growing interest in epigenetic variation influenced by environmental exposures (i.e. non-genetic factors) in the molecular etiology of psychiatric disorders [26]. While most gene variants identified with genome-wide association studies (GWAS) do not directly code for changes affecting protein structure and function [27], it was recently shown that many of the SZ GWAS loci overlap with DNAm modifications associated with SZ [9]. Thus, DNAm can be influenced by both genetic and environmental factors and can contribute to the regulation of gene expression of GWAS loci associated with psychiatric disorders [9].

As part of the response to hypoxia, recent findings have identified modifications in DNAm, which reflect a regulation in the expression and function of several important genes including membrane receptors, ion channels, key enzymes and signaling proteins in fetal organs and tissues [23]. Given that DNAm is sensitive to environmental factors and the response to PA is usually systemic, DNAm changes in peripheral tissues can represent an important molecular marker to study the role of the response to PA in mental disorders.

The aim of the current study was to assess the putative interactions between DNAm profiles and PA, a variable obtained from prospective birth registry data, in association with case-control status. We hypothesize that PA is associated with divergent epigenetic modifications in individuals with SZ or BD compared to healthy controls.

## Materials and methods

### Participants

The current study used data from 598 individuals with SZ (*n* = 388) or BD (*n* = 210) from the Thematically Organized Psychosis (TOP) study, the main study protocol of the Norwegian Centre for Mental Disorders Research (NORMENT; Oslo, Norway), 14 individuals with SZ from the Youth Thematic Organized Psychosis (Youth-TOP; Oslo, Norway) study and 31 individuals with SZ (*n* = 28) or BD (*n* = 3) from the Early Diagnostic and Treatment of Psychosis (TIPS; Stavanger, Norway) study. The 676 healthy controls (HC) from the TOP or Youth-TOP study were recruited from the greater Oslo area based on random selection from the Norwegian national population registry. Participants were of European ancestry, between the ages of 13–49 years (median age = 29 years) and 45% female (589/1319). All participants gave written informed consent. The study was conducted in accordance with the Helsinki Declaration with approval from the Regional Committees for Medical Research Ethics South East Norway (REC South East) and the Norwegian Data Inspectorate.

Between October 2002 and 2013, adult TOP and adolescent Youth-TOP individuals with SZ and BD spectrum disorders, respectively, were consecutively recruited from psychiatric units (outpatient and inpatient units) of public hospitals in the Oslo region and Stavanger area for the TIPS participants. All participants with SZ and BD underwent thorough clinical investigation by trained psychologists and physicians. Blood samples were drawn from fasting participants and within a narrow time window in the morning. Clinical diagnoses were assessed using the Structured Clinical Interview for DSM-IV axis 1 disorder (SCID-I) module A-E [28]. For the Youth-TOP sample, we used the Norwegian version of the Kiddie-Schedule for Affective Disorders and Schizophrenia for School Aged Children (6–18 years): Present and Lifetime Version (K-SADS-PL) [29]. Psychosocial function was assessed with the Global Assessment of Functioning scale, split version (GAF) [30]. Current psychotic symptoms were rated by the use of the Positive and Negative Syndrome Scale (PANSS) [31]. The Alcohol Use Disorders Identification Test (AUDIT) [32] and Drug Use Disorders Identification Test (DUDIT) [33] were used to evaluate alcohol and drug use. HC were interviewed by trained research assistants and examined with the Primary Care Evaluation of Mental Disorders (Prime-MD) to ensure no current or previous psychiatric disorders [34].

Exclusion criteria for both patients (PT) and HC were organic disorders (substance-induced psychotic disorder, somatic health condition, brain damage or head trauma with unconsciousness over 5 min, neurological diseases and autism spectrum disorder). Additional exclusion criteria for HC were current or previous somatic illness and substance misuse disorders or dependency within the last 6 months. HC were excluded if they or a first-degree relative had a lifetime history of a severe psychiatric disorder. Additionally, TOP and Youth-TOP participants underwent magnetic resonance imaging (MRI), and scans were assessed by a neuroradiologist using a graded scheme (See TOP MRI Grading Scheme) [35]. If brain pathology was detected, the participant was also excluded.

**Fig. 1:**
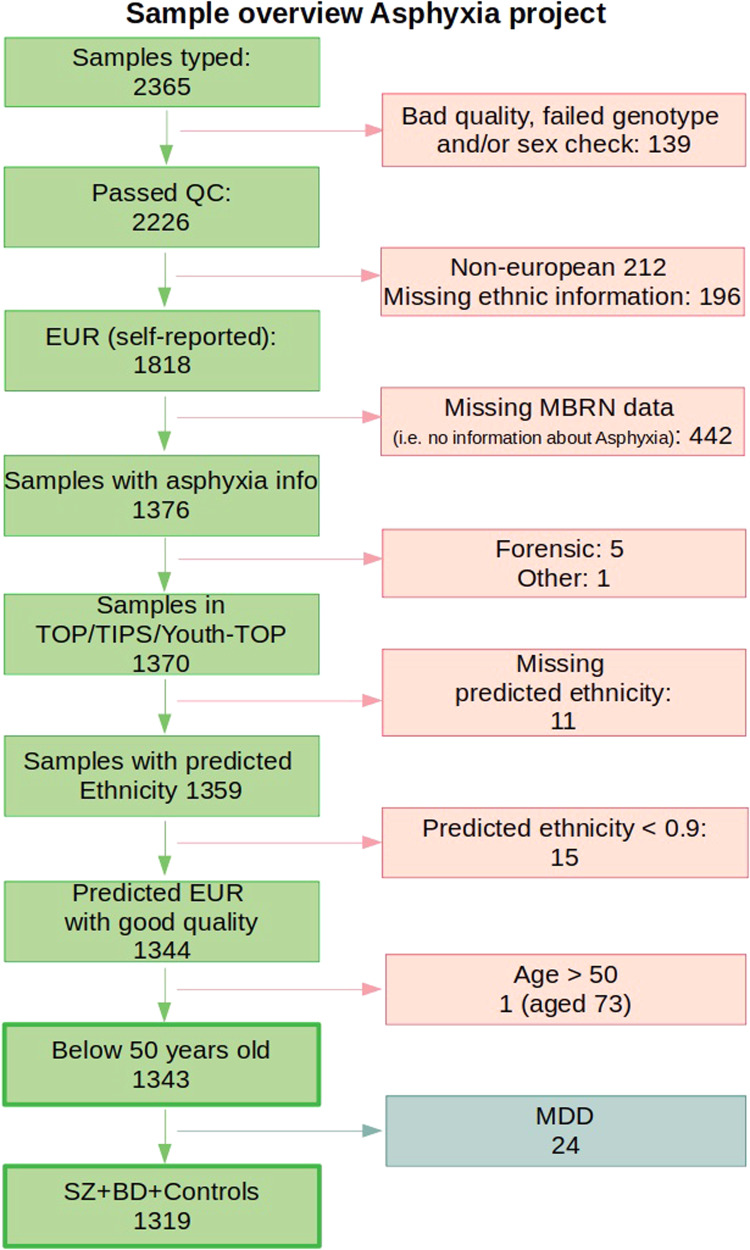
Overview of the participants included in the sample. Perinatal asphyxia data from the Medical Birth Registry of Norway (MBRN) is available on births from 1967; therefore, if the participant was not born in Norway or is >50 years, they would not have birth registry information in the MBRN. Quality control (QC); European ancestry (EUR); schizophrenia (SZ); bipolar disorder (BD); major depressive disorder (MDD).

A flow diagram of the selection of participants for this study is found in Fig. 1. Individuals with one of the following DSM-IV diagnoses were included in this study: [1] within the *SZ spectrum*, *n* = 430 [schizophrenia (DSM-IV 295.1, 295.2, 295.3, 295.6, and 295.9; *n* = 254), schizophreniform disorder (DSM-IV 295.4; *n* = 29), and schizoaffective disorder (DSM-IV 295.7; *n* = 48) or other psychosis (psychosis not otherwise specified, DSM-IV 298.9, *n* = 72; brief psychotic disorder, DSM-IV 298.8, *n* = 7; delusional disorder, DSM-IV 297.1, *n* = 20)] and [2] within the *BD spectrum*, *n* = 213 [Bipolar I disorder (DSM-IV 296.0–7; *n* = 133), Bipolar II disorder (DSM-IV 296.89; *n* = 63) or bipolar disorder not otherwise specified (DSM-IV 296.80; *n* = 17)].

### Perinatal asphyxia (PA)

The birth data was collected from the Medical Birth Registry of Norway (MBRN), which has mandatory reporting on all births after 16-weeks gestational age. The PA variable included complications coded AS53 (asphyxia without other signs), AS54 (asphyxia with poor sound), AS55 (asphyxia and discolored amniotic fluid), AS61 (asphyxia), P211 (mild or moderate birth asphyxia) in the registry.

### DNAm analysis

Methylation quantification was completed using the Illumina Infinium® Methylation EPIC BeadChip (Illumina, Inc. San Diego, USA).

### Pre-processing and quality control (QC)

Epigenome-wide DNA methylation was measured in a total of 2365 blood samples. The measurements were performed in three batches of 1000, 283 and 1082 samples, respectively. Quality control (QC) and preprocessing were performed on the data from each batch separately using the following pipeline: (1) removal of sites with a detection *p*-value > 0.01 in at least 1% of the samples, and removal of samples where at least 1% of the sites had a detection *p*-value > 0.01, together with removal of sites with a bead count <3 in 5% of the samples; (2) removal of problematic probes that are known to cross-hybridize or contain single nucleotide polymorphisms (SNPs) that are close to target cytosine-phosphate-guanine sites (CpGs) [36]; (3) samples from individuals that were predicted to be of European ancestry with a probability of <0.9 were removed. Ancestry was predicted from genetic data, using a Random Forest classifier. Populations from the 1000 genome project were used to train the model; (4) functional normalization of the data was performed to reduce non-biological variation. The number of principal components (PCs) used in the normalization was 20 PCs for the first and second batches, and 25 PCs for the third batch; (5) removal of samples with a mismatch between SNP genotypes from the EPIC array and genotype data from the same samples; (6) removal of samples where the predicted and the reported sex did not match; (7) removal of sex chromosome probes; and (8) removal of batch effects from AMP plate, Sentrix ID, Sentrix Position, run date and scanner ID using ComBat [37] as implemented in the bioconductor package sva. The statistical programming software R (version 4.0.0) and Bioconductor (version 3.1.1) packages minfi, wateRmelon, sva and ChAMP were used during the QC and preprocessing of the data. After the QC and preprocessing procedures, the data from the three batches were merged, and ComBat was used to remove the batch effects.

Finally, the subset of samples for which information about PA status is available was extracted and used for the analyses in this study. The final dataset had 1319 samples and 760668 probes.

#### Epigenome wide association study (EWAS)

We performed an epigenome wide association study (EWAS) to identify differentially methylated positions (DMPs) associated with PA. Because cigarette smoking and white blood cell composition can affect methylation [9], smoking scores and white blood cell ratios were used as covariates in analyses, together with age and sex. The smoking scores were calculated using a method described by Elliott and colleagues [38]. The blood cell ratios were calculated using the estimateCellCounts2 function from the bioconductor package, FlowSorted.Blood.EPIC. Differentially methylated regions (DMRs) were identified using the *comb-p* algorithm [34], specifying parameters as seed *p*-value = 0.05 and maximum distance between probes of 750 base pairs, as recommended by Mallik and colleagues [39]. The *comb-p* algorithm was also run with the more stringent seed *p*-value = 0.001. Šidák correction was used to account for multiple testing. Consistent with previous studies, a significance threshold was set to false discovery rate (FDR) < 0.05 for DMPs or Šidák *p* < 0.05 for DMRs [37]. DMRs were considered significant with three or more probes and a Šidák *p* < 0.05. DMPs and DMRs were annotated using the bioconductor package IlluminaHumanMethylation EPICanno.ilm10b4.hg19 [35].

Because we were interested in whether DNAm in blood is altered in response to PA and the relevance these alterations might have for associated loci in the brain, we used the online Blood Brain DNA Methylation Comparison Tool [40]. This online tool is a searchable database containing correlations between DNAm in blood and four brain regions (prefrontal cortex, entorhinal cortex, superior temporal gyrus and cerebellum) for each of the CpGs that are included on the Illumina 450 K DNA methylation chip.

#### Other statistical analyses

As we hypothesized a different response to PA in cases and controls, we used linear models to evaluate the interaction of Blood methylation status (M-values) [41] and PA on case–control status (Dx ~ M-Values*PA + covariates). Initial analyses with age, sex, smoking score and blood cell ratios as covariates inflated the QQ-plots. The first 10 principal components calculated from the DNAm data were therefore also added as covariates, which effectively dampened the inflation. Nominal *p*-values were converted to FDR values following the Benjamini and Hochberg approach [42]. We used summary statistics from the EWAS and Pearson’s correlation to assess the relationship of PA related DNAm between groups [43]. We re-ran significant analyses stratified by sex or patient subgroups. We also explored the relationship between M-values and PA on age of onset and current symptom severity (total PANSS score) using the model: age of onset or severity ~ M-Values*PA + covariates, stratified by sex (males/females only).

## Results

PT and HC differed in age by 3 years (mean age of 28 and 31 years, respectively; *F* = 57.54, *p* < 0.001), but not in the distribution of sex (47 and 43% female; *χ*^2^ = 1.73, *p* = 0.19). Both age and sex were included as covariates in the analyses. The incidence of PA was also similar 12% in the PT group and 15% in the HC group (*χ*^2^ = 2.52, *p* = 0.13). Further, demographic and clinical data are presented in Table 1.

**Table 1:** Demographic and clinical characteristics.

									Pairwise	
	PT (*n* = 643)			HC (*n* = 676)			Main effect of Group		PT vs. HC	
	PA-	PA+		PA-	PA+		Statistic (df)	*p*	Mean difference (SE)	*p*
PA, *n* (% of total sample)	566 (88)	77 (12)		576 (85)	100 (15)		*χ*^2^ = 2.52 (1, 1319)	0.13		–
SZ, *n* = 430 (% of sub sample)	386 (90)	44 (10)					*χ*^2^ = 4.83 (1, 1106)	0.03	(HC > SZ)	
BD, *n* = 213 (% of sub sample)	180 (85)	33 (16)					*χ*^2^ = 0.06 (1, 889)	0.80		–
			F /*χ*^2^ (df)			F /*χ*^2^ (df)				
Mean age, years (SD)	28.10 (6.95)	28.35 (6.94)	0.09 (1, 641)	31.28 (7.39)	30.22 (7.10)	1.78 (1, 674)	*F* = 57.54 (1, 1317)	< 0.001	-2.99 (0.39)	< 0.001
Sex, M/ F (% Males)	302/ 264 (53)	42/ 35 (55)	0.04 (1, 643)	319/ 257 (55)	67/ 33 (67)	*4.70 (1, 676)	*χ*^2^ = 1.73 (1, 1319)	0.19		–
Handedness, No. non-right (% non-right)	68/ 499 (14)	10/ 66 (15)	0.11 (1, 565)	61/ 566 (11)	7/ 98 (7)	1.20 (1, 664)	*χ*^2^ = 3.71 (1, 1229)	0.05		
							FET	0.03		(PT > HC)
Tobacco use, Yes/ No (% Yes)	317/ 225 (59)	45/ 30 (60)	0.06 (1, 617)	87/ 179 (33)	16 / 35 (31)	0.04 (1, 317)	*χ*^2^ = 57.41 (1, 934)	< 0.001		(PT > HC)
AUDIT, score (SD)	8.13 (7.06)	7.35 (5.81)	0.61 (1, 501)	5.66 (3.38)	5.33 (3.08)	0.55 (1, 486)	F = 43.05 (1, 971)	< 0.001	2.27 (0.35)	<0.001
DUDIT, score (SD)	4.30 (7.63)	2.64 (5.27)	2.45 (1, 497)	0.29 (1.42)	0.37 (1.44)	0.21 (1, 488)	*F* = 124.96 (1, 970)	< 0.001	3.78 (0.34)	<0.001
Adult height, cm (SD)^a^	175.21 (6.52)	174.79 (6.53)	0.27 (1, 597)	176.76 (5.95)	174.45 (5.95)	*5.33 (1, 288)	*F* = 0.72 (1, 888)	0.40	-0.39 (0.46)	0.40
Adult weight, kg (SD)^a^	79.71 (17.84)	80.53 (17.34)	0.06 (1, 273)	76.00 (12.47)	75.60 (12.49)	0.03 (1, 163)	*F* = 7.47 (1, 439)	0.007	4.33 (1.59)	0.007
GA, weeks (SD)	39.77 (1.93)	39.34 (3.66)	2.39 (1, 606)	39.82 (1.74)	39.98 (2.48)	0.56 (1, 637)	*F* = 1.25 (1, 1245)	0.26	-0.13 (0.12)	0.26
GA (36–37 weeks), No. (%)	30/ 535 (6)	3/ 73 (4)	0.28 (1, 608)	35/ 544 (6)	7/ 95 (7)	0.12 (1, 639)	*χ*^2^ = 0.00 (1, 1247)	0.97		–
GA (34-35 weeks), No. (%)	10/ 535 (2)	0/ 73 (0)	1.39 (1, 608)	6/ 544 (1)	4/ 95 (4)	*5.07 (1, 639)	*χ*^2^ = 0.01 (1, 1247)	0.91		–
GA (<33 weeks), No. (%)	6/ 535 (1)	8/ 73 (11)	**27.63 (1, 608)	3/ 544 (1)	2/ 95 (2)	2.52 (1, 639)	*χ*^2^ = 4.80 (1, 1247)	0.03		(PT > HC)
BW, gram (SD)^a^	3541 (555)	3282 (555)	**14.74 (1, 640)	3533 (555)	3506 (557)	0.20 (1, 672)	F = 0.22 (1, 1315)	0.64	-14.54 (30.75)	0.64
BW (2000-2500 g), No. (%)	10/ 566 (2)	3/ 77 (4)	1.55 (1, 643)	12/ 575 (2)	4/ 100 (4)	1.35 (1, 675)	*χ*^2^ = 0.19 (1, 1318)	0.67		–
BW (1500-1999 g), No. (%)	4/ 566 (1)	4/ 77 (5)	**11.11 (1, 643)	6/ 575 (1)	3/ 100 (3)	2.48 (1, 675)	*χ*^2^ = 0.02 (1, 1318)	0.89		–
BW ( < 1499 g), No. (%)	0/ 566 (0)	3/ 77 (4)	**22.16 (1, 643)	0/ 575 (0)	1/ 100 (1)	*5.76 (1, 675)	*χ*^2^ = 1.10 (1, 1318)	0.29		–
Apgar 1 min 0–3, No. (%)	0/ 387 (0)	3/ 56 (5)	**20.87 (1, 443)	0/ 354 (0)	4/ 70 (6)	**20.42 (1, 424)	*χ*^2^ = 0.19 (1,867)	0.66		
Apgar 1 min 4–7, No. (%)	23/ 387 (6)	24/ 56 (43)	**70.29 (1, 443)	13/ 354 (4)	23/ 70 (33)	**64.07 (1, 424)	*χ*^2^ = 1.12 (1,867)	0.29		
Apgar 5 min 0–3, No. (%)	0/ 385 (0)	0/ 55 (0)		0/ 355 (0)	1/ 70 (1)	*5.08 (1, 425)	*χ*^2^ = 1.04 (1,865)	0.31		
Apgar 5 min 4–7, No. (%)	1/ 385 (0)	8/ 55 (15)	**49.02 (1, 440)	1, 355 (0)	7/ 70 (10)	**29.90 (1, 425)	*χ*^2^ = 0.03 (1,865)	0.86		
GAF-S, score (SD)	47.29 (13.65)	49.11 (12.23)	1.21 (1, 626)							
GAF-F, score (SD)	48.35 (13.32)	48.51 (13.08)	0.01 (1, 621)							
PANSS, score (SD)	57.28 (16.99)	56.34 (13.89)	0.21 (1, 623)							
General, score (SD)	30.26 (8.45)	30.04 (7.10)	0.05 (1, 625)							
Negative, score (SD)	13.62 (5.89)	13.09 (5.11)	0.56 (1, 626)							
Positive, score (SD)	13.42 (5.35)	13.21 (4.92)	0.10 (1, 628)							
Medication, *n* (% of sample)										
Antipsychotics	401 (71)	49 (64)	1.35 (1, 643)							
Antidepressants	169 (30)	18 (23)	1.08 (1, 643)							
Antiepileptics	115 (20)	17 (22)	0.04 (1, 643)							

*PT* patients, *HC* healthy controls, SZ schizophrenia spectrum, *BD* bipolar disorder spectrum, *PA−* without exposure to perinatal asphyxia, *PA+* with exposure to perinatal asphyxia, *FET* Fisher’s Exact Test, *SD* standard deviation, *SE* standard error of the mean, *GA* gestational age, *BW* birth weight, *AUDIT* Alcohol Use Disorders Identification Test, *DUDIT* Drug Use Disorders Identification Test, *GAF-S* Global Assessment of Functioning scale, split version, symptoms, *GAF-F* Global Assessment of Functioning scale, split version, functioning, *PANSS* Positive and Negative Syndrome Scale.

*p < .05, **p ≤ .001

^a^Corrected for sex

### Genome-wide identification of differentially methylated positions

No significant associations were found for any of the models (EWAS or interaction analysis) at the single position level, after correction for multiple testing. A Manhattan plot for the main effect of PA is presented in the SI, Supplementary Fig. 1.

### Genome-wide identification of differentially methylated regions

**Fig. 2:**
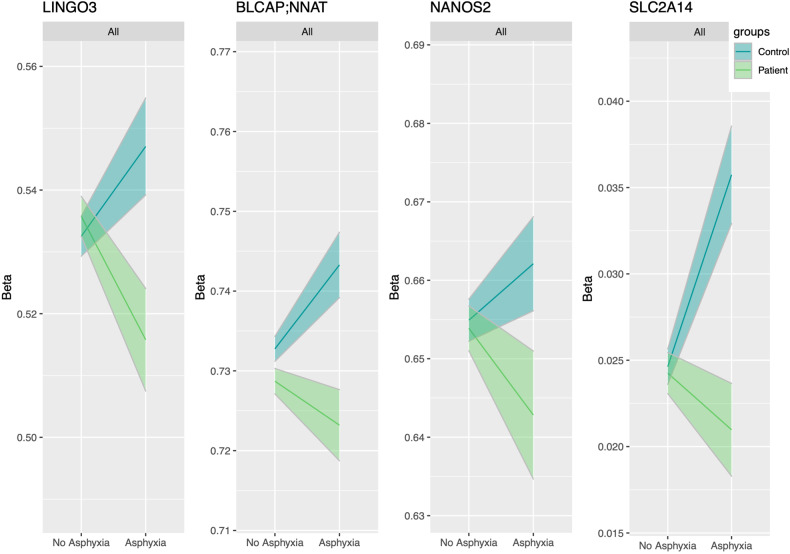
Differentially methylated regions (DMRs) with a significant perinatal asphyxia × DNAm interaction on case/control status. Across the four DMRs, methylation patterns differed between groups with perinatal asphyxia exposure. Shaded area represents standard error of the mean (SE). Beta values are for blood methylation status (y-axis).

Significant interactions between four DMRs and PA on case-control status were identified in genes encoding leucine-rich repeat and immunoglobulin-like domain-containing nogo receptor-interacting protein 3 (*LINGO3*, Šidák corrected *p* = 1.71E-03), the overlapping bladder cancer-associated protein and neuronatin (*BLCAP; NNAT*, Šidák corrected *p* = 4.88E-03), nanos C2HC-type zinc finger 2 (*NANOS2*, Šidák corrected *p* = 6.92E-03) and solute carrier family 2 member 14 protein (*SLC2A14*, Šidák corrected *p* = 3.95E-02; Fig. 2 and Table 2), so that HC with PA had higher methylation at these DMRs compared with HC without PA, while the opposite was the case in patients (PT). From another point of view, HC and PT had different methylation at these DMRs if they had a history of PA exposure, with HC having higher methylation compared with PT (Fig. 2).

**Table 2:** Significant interaction between differentially methylated regions (DMRs) and perinatal asphyxia on Case/Control status.

Šidák *p* < 0.05		PA* DNAm			PA			DNAm		
DMR	*gene*	Min *p*	n probes	Šidák *p*	Min *p*	n probes	Šidák *p*	Min *p*	n probes	Šidák *p*
chr19:2291373-2291613	*LINGO3*	5.97E-04	3	1.71E-03						
chr20:36148615-36149022	*BLCAP;NNAT*	2.92E-02	19	4.88E-03						
chr19:46417551-46417734	*NANOS2*	3.87E-02	4	6.92E-03						
chr12:8025435-8025646	*SLC2A14*	1.63E-02	5	3.95E-02	6.59E-03	3	1.53E-02			
chr2:242802009-242802192	*PDCD1*							4.09E-09	5	1.12E-10
chr4:38525620-38525916	*LINC01258*							9.43E-07	3	2.49E-08
chr19:37825211-37825679	*HKR1*							1.91E-06	10	5.98E-08
chr3:45077254-45077881	*CLEC3B*							7.48E-03	4	5.93E-06
chr19:58220494-58220955	*ZNF154*							4.36E-04	8	3.24E-05
chr4:1243849-1244086	*CTBP1;C4orf42*							7.17E-04	6	1.27E-04
chr7:2316636-2317124	*SNX8*							1.29E-03	9	1.27E-04
chr19:1622838-1623075	*TCF3*							1.43E-03	3	2.35E-04
chr11:57232407-57232556	*RTN4RL2*							1.19E-03	3	2.40E-04
chr6:30653407-30653732	*KIAA1949*							1.19E-03	6	3.09E-04
chr2:234359654-234359783	*DGKD*							1.29E-03	3	3.61E-04
chr1:24645802-24646205	*GRHL3*							1.90E-03	11	6.20E-04
chr20:39766765-39766794	*PLCG1-AS1;PLCG1*							8.25E-04	3	6.54E-04
chr8:1765066-1765477	*MIR596*							3.85E-03	9	6.85E-04
chr11:43942418-43942604	*ALKBH3-AS1*							3.48E-03	4	1.05E-03
chr8:144222103-144222455								4.96E-03	4	1.18E-03
chr8:143859669-143859906	*LYNX1*							3.47E-03	7	1.46E-03
chr4:206112-206339	*ZNF876P*							7.21E-04	6	3.14E-03
chr1:949392-949850	*ISG15*							1.13E-02	4	3.44E-03
chr8:23563859-23564193	*NKX2-6*							9.85E-03	8	4.45E-03
chr1:3600735-3600879	*TP73*							7.37E-03	3	4.67E-03
chr11:118758504-118758721	*CXCR5*							1.05E-02	4	5.36E-03
chr5:150326497-150326642	*LOC134466*							2.64E-03	3	5.68E-03
chr6:31846769-31847028	*SLC44A4*							1.16E-02	7	6.35E-03
chr17:75451669-75451846	*SEPT9*							1.16E-02	4	9.39E-03
chr17:79817079-79817271	*P4HB*							1.48E-02	4	1.28E-02
chr3:10326540-10326774	*GHRLOS;C3orf42*							1.87E-02	3	1.50E-02
chr21:42741788-42741991	*MX2*							7.37E-03	4	1.57E-02
chr17:3561358-3561497	*CTNS*							1.16E-02	3	1.61E-02
chr1:234871253-234871477								1.95E-02	3	1.71E-02
chr17:42145037-42145270	*LSM12*							2.28E-02	3	1.99E-02
chr1:2949633-2949673								9.06E-03	3	2.33E-02
chr12:10563947-10564015	*KLRC4-KLRK1*							1.16E-02	3	2.35E-02
chr12:117470875-117471115								2.96E-02	4	3.01E-02
chr12:131322960-131323042	*STX2*							1.53E-02	3	3.19E-02
chr13:113655964-113656212	*MCF2L*							1.16E-02	7	3.25E-02
chr19:8274853-8275083	*LASS4*							2.03E-03	3	3.69E-02
chr6:31508106-31508318	*BAT1*							1.17E-02	6	3.70E-02
chr12:132340356-132340570								3.21E-02	4	3.88E-02
chr2:204801413-204801510	*ICOS*							1.95E-02	4	3.90E-02

Positions of DMRs are given according to hg19 reference genome with chromosome: start-end. DMRs were identified using a seed with a *p-*value of < 0.05. For each DMR, the table lists the minimum *p*-value (Min *p*), the number of probes (n probes) and the Šidák corrected *p*-value (Šidák *p*). *PA* perinatal asphyxia

**Fig. 3:**
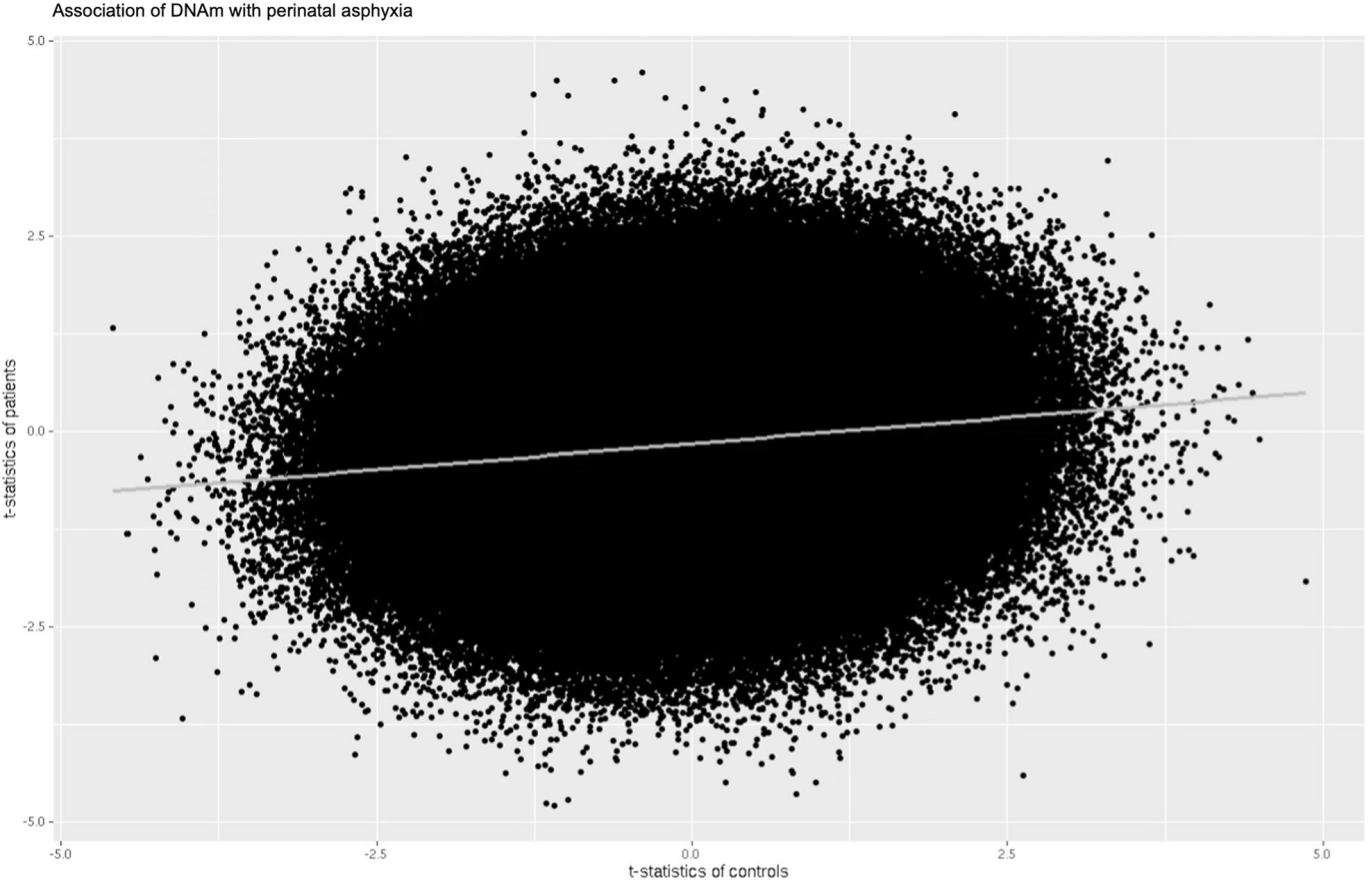
Scatterplot demonstrating the correlation between DNA methylation profiles associated with perinatal asphyxia between groups.

We analyzed the correlation of DNAm profiles associated with PA between PT and HC, to explore whether the interaction detected at the DMR in *LINGO3, BLCAP; NNAT, NANOS2 and SLC2A14* may reflect a global pattern of opposite response to PA. We observed a weak, albeit significant, positive correlation of DNAm profiles associated with PA between PT and HC, *r* = 0.134, *p* < 2.2e-16 (Fig. 3). Stratified by sex, we found a weak negative correlation of DNAm profiles associated with PA between female PT and HC (*r* = -0.120, *p* < 2.2e-16) and a weak positive correlation between male PT and HC (*r* = 0. 088, *p* < 2.2e-16; Supplementary Fig. 2).

We found significant correlations between methylation in blood and methylation in previously tested brain regions, namely prefrontal cortex, entorhinal cortex, and superior temporal gyrus, for probes associated with *LINGO3, BLCAP;NNAT, NANOS2* and *SLC2A14* (Supplementary Figs. 3–6).

### Sex, PA and differentially methylated regions

In males, we found significant interactions between DMRs and PA on case-control status in six genes encoding *TNFAIP8, ADARB2, SLC35C1, SORBS2, NRIP2* and *MOBP* (Supplementary Table 1). In females, we identified one gene, *DUSP19*, with a significant interaction between DMRs and PA on case-control status (Supplementary Table 2).

### Differentially methylated regions and PA on Age of onset or disease severity

One DMR for the gene encoding *TACSTD2* in male PT, and one in female PT**—***LGALS8*, had a significant interaction between PA and methylation on disease severity (Supplementary Table 3). In male PT, we identified one DMR for the gene encoding *TMEM232* that showed a significant interaction between PA and methylation on age of onset (Supplementary Table 4). No associations between PA and methylation on age of onset were observed in female PT.

### Differentially methylated regions and PA on SZ and BD subgroups

Significant interactions between six DMRs and PA on SZ case-control status were identified for the *HOXA4, SLC2A14, CTBP1;C4orf42, MOBP, BAHCC1 and NANOS2* genes (Supplementary Table 5). Significant interactions between two DMRs and PA on BD case-control status were identified for the *HKR1 and LRRC34* genes (Supplementary Table 6). We observed a significant positive correlation of DNAm profiles associated with PA between SZ and BD, *r* = 0.295, *p* < 2.2e-16 (Supplementary Fig. 7).

## Discussion

By analyzing the interaction between DNAm and PA on case-control status, we identified DMRs in genes encoding *LINGO3, BLCAP;NNAT, NANOS2* and *SLC2A14*. In the absence of PA, PT and HC have the same methylation level; while PA exposure corresponded to an increase of methylation in HC and a decrease of methylation in PT. Consistently, we found a weak global correlation in methylation profiles associated with PA between groups.

The increase of methylation at these DMRs in HC may be part of an adaptive epigenetic response to PA, which could be absent in PT, who indeed have lower DNAm at these DMRs, in the context of PA. PA may induce different responses in the epigenome, and some of these paths may increase the risk for psychiatric disorders. Since previous studies have found alterations with *LINGO3* [44–47]*, BLCAP;NNAT* [48–51] *and SLC2A14* [52, 53] genes or associated functions in SZ and BD, it might be possible that an altered response to PA at the DNAm and/or gene expression levels may disrupt brain development and function in PT. DNAm increases in HC might represent epigenetic modifications influenced by environmental exposure of PA, which may be associated with increased susceptibility to somatic diseases later in life, but not necessarily the transition to severe mental disorders. It may also represent protective-adaptive alterations that may increase resilience in healthy individuals later in life.

Due to its availability and ease of collection, this EWAS used blood as a surrogate tissue rather than disease specific tissue; however, methylation levels in blood and brain-tissue have a concordance [54]. To further address this issue, we performed secondary analyses on the probes associated with *LINGO3, BLCAP;NNAT, NANOS2 and SLC2A14* to investigate the relationship between DNAm in blood and at the same genomic loci in four brain regions (prefrontal cortex, entorhinal cortex, superior temporal gyrus and cerebellum) [40] and found significant correlations. However, because the response to PA is systemic and given the stability of DNAm profiles, our findings have relevance to psychiatric disorder even in the absence of a proven brain-blood correlation. In other words, changes in DNAm detected in blood may reveal an epigenetic mechanism of response to PA which is relevant for the development of psychiatric disorders.

Nonetheless, we detected significant interactions between PA and DNAm at the level of DMR mapping to loci containing genes with relevant brain functions. Particularly, *LINGO3*, a paralog to *LINGO1*, is expressed in neurons and a highly restricted population of Olig2-expressing oligodendroglia cells. A functional overlap between *LINGO3* and *LINGO1* has been suggested [55]. The more studied, *LINGO1*, is a potent negative regulator of neuron and oligodendrocyte survival, axon regeneration, neurite extension, oligodendrocyte differentiation, axonal myelination and functional recovery; these processes are highly involved in numerous brain functions [56]. Reduced oligodendrocyte densities have been reported in both SZ and BD [44–47]. In SZ, *LINGO1* has been found to be upregulated in the dorsolateral prefrontal cortex and hippocampus [57] and associated with the dysregulation in apoptosis of neurons [58], which might explain previously observed reductions in mean total neuron number in the putamen and caudate nucleus of individuals with SZ [59]. So, if the DNAm changes that we detected in blood are mirrored by DNAm changes in the brain, as our results suggest, we could speculate that PA may lead to a decrease of methylation in individuals who later in life developed SZ or BD. Such DNAm decrease may be associated with an increased *LINGO3* expression, consistent with the findings of increased *LINGO1* expression and reduced oligodendrocyte densities in SZ and BD.

For the overlapping *BLCAP;NNAT* genes, the *BLCAP* gene encodes a protein that reduces cell growth by stimulating apoptosis, and the imprinted gene *NNAT* encodes a protein associated with brain development, assembly, maturation and maintenance of the central nervous system (CNS) [60]. A recent EWAS study found that the DNAm of *BLCAP;NNAT* were strongly associated with left-handedness [61], a trait with low heritability and where epigenetic mechanisms have been proposed as an underlying etiological mechanism. Numerous scientific studies have demonstrated a higher occurrence of left-handedness in an array of psychiatric disorders, supporting the view that there is a genetic link between handedness and brain lateralization [48–51]. It is also known that left-handedness is associated with complications in the perinatal period [48, 62]. Consistent with these studies, we report here a higher frequency of non-right handers among PT who experienced PA compared to PA in the HC.

Of note, *NNAT* is an imprinted gene [60], and alterations in the DNAm of imprinted genes (i.e. genes that carry parental allele-specific methylation profiles) have been documented in studies of *in utero* exposure to dietary micronutrients [63, 64], caloric restriction [65–67], protein restriction [68] and cigarette smoking [69]. The PA related differences in the DNAm of the *NNAT* gene reported here may be an example of a methylation alteration in an imprinted gene that can serve as a useful biosensor of adverse environmental exposures that also include PA [70].

*NANOS2* and *Nanos* genes are expressed in fetal and adult testis and ovary and the adult brain, particularly the hippocampus [71], and are known for their evolutionarily preserved role in germ cell pluripotency and survival [72]. Nanos proteins are important in the development of the CNS [71, 73], but also their overexpression is associated with various human cancers [72, 74], consistent with the link between cancer and gene regulatory network acting during development. Specifically, alterations due to DNAm of *Nanos* genes have been associated with hepatocellular carcinoma [75], prostate cancer [76], thyroid carcinomas [77], adult [78] and childhood [79] asthma, type II diabetes [80] and metabolic syndrome [81]. Of interest, an animal study has detected increased *NANOS2* expression in the hypothalamus following exposures to maternal deprivation, consistent with the possibility that other early life stressors, in addition to PA, may alter the epigenetic status of this gene, whose function as a zinc finger protein may contribute to regulate the translation of genes relevant for development [82].

*SLC2A14* gene, a paralog of *SLC2A3*, is highly expressed in the brain and other tissues: cardiomyocytes, placenta, white blood cells, and others [83]. In both the neocortex and in deeper cortical structures, *SLC2A3* seems to be primarily localized to axonal and dendritic processes, suggesting an important role for *SLC2A3* in ATP‐dependent axonal transport processes and synaptic plasticity [84]. Furthermore, *SLC2A3* has been shown to strongly respond to hypoxic stress [85] and it has been shown that hypoxia-induced chromatin conformation changes influence *SLC2A3* expression and functions in multiple cell types [86]. Consistent with our findings, it has been suggested that alterations in *SLC2A3* gene dosage interfere with neurodevelopment of individuals later diagnosed with a neuropsychiatric disorders, such as ADHD [87], SZ [52] and BD [53].

A strength of this study is the prospective birth registry information on PA that was available on a large sample of individuals with SZ or BD and HC. A limitation to the study is represented by our sample size, which reduces the power to detect changes at the differentially methylated position level. Stratifying by sex reduced the sample size considerably, so we interpret these findings and associations with disease severity and age of onset with caution.

Another limitation to this study is that we define PA based on codes from the MBRN, which may not fulfill modern criteria for PA [88]. Indeed, most of the participants were born before 1990, thus before the definition of current criteria for PA [88]. Additionally, the use of encoded data from the MBRN may not accurately describe the clinical picture of the condition and may account for the over-registration of PA (>10%) in this study. However, given that MBRN data are collected from maternity wards in Norwegian hospitals, we can assume that the conditions classified as PA in the MBRN (asphyxia without other signs, asphyxia with poor sound, asphyxia and discolored amniotic fluid, asphyxia, mild or moderate birth asphyxia) were defined based on the ICD classification of asphyxia (e.g., “failing to initiate and sustain breathing at birth”), which should also capture more recent strict definition of this important complication. Inflammatory presentation is unclear in the MBRN. Since inflammation together with PA increases the risk for neural damage [89], milder insults may cause injury or alterations in those with a genetic liability for SZ development [90]. Given that PA is associated with neurodevelopmental deficits later in life, the exclusion criteria of the TOP study (i.e., exclusion based neurological diseases and intellectual impairments) might not fully capture the full range of PA exposure in our patient sample. Additionally, the exclusion criteria might be the reason for the lower percentage of PA in the SZ group, which have a higher genetic liability for SZ but may also have a lower tolerance for PA.

Since common genomic variants and epigenetic modifications influenced by environmental risk factors are frequent in a population and in part shared with other psychiatric disorders [91], future studies might include larger and more diverse cohorts of mental and neurodevelopmental disorders to investigate the specificity of these findings. Given the polypharmacy among patients, a potential effect of medication was unavoidable and is a limitation of any clinical study, including ours. Future studies addressing the influence of psychiatric medication and simultaneous use of several psychiatric medications on DNAm, also in combination with early life complications, might be important. Since PA commonly co-occurs with other severe obstetric complications [35], we cannot rule out that other underlying conditions like placental pathophysiology, in which placental failure can lead to asphyxia and subsequent growth restriction or preterm births [92] (see Supplementary Note 1), might influence DNAm in our sample. Additionally, possible maternal risk factors, such as preeclampsia, maternal diabetes and BMI, could influence developmental outcomes in offspring and may explain both the DMRs as well as later PT status. Future work in larger samples should include additional environmental risk factors like maternal and placental conditions and exposures in combination with DNAm and offspring patient status to better define what may drive the differential methylation in response to PA.

We identified four DMRs for *LINGO3, BLCAP;NNAT, NANOS2 and SLC2A14* genes that significantly interacted with PA on case-control status, where methylation patterns differed with PA exposure between groups. These findings provide further evidence of alterations in the response to hypoxia and oxidative stress and may underscore the contribution of PA as a source of the shared etiopathogenetic mechanisms leading to the abnormal brain development commonly observed in schizophrenia and bipolar disorder. Prevention strategies may benefit from understanding the possible divergent epigenetic responses to PA that give rise to a healthy brain development or to trajectories of risk for psychiatric disorders. We mainly assessed DNAm in adults but examining infants or young children might be of interest for future studies aimed at detecting epigenetic changes specific to early stages of development, which could represent biomarkers of risk.

### Supplementary information

Supplemental material

## Data Availability

The datasets used and/or analyzed during the current study are available from the corresponding author on reasonable request. The data are not publicly available due to privacy or ethical restrictions.
