# Supplementary material for: Divergent epigenetic responses to perinatal asphyxia in severe mental disorders
Source: Transl Psychiatry. 2024 Jan 8;14:16. doi: 10.1038/s41398-023-02709-7 (PMC10774425; doi:10.1038/s41398-023-02709-7)
Supplement: Supplementary file 1 — Supplemental material [file 41398_2023_2709_MOESM1_ESM.docx]

**Divergent epigenetic responses to perinatal asphyxia in severe mental disorders**

Laura A. Wortinger, PhD ^1, 2 *^ ^#^; Anne-Kristin Stavrum, PhD ^3, 4 #^; Alexey A. Shadrin, PhD ^2, 5, 6^; Attila Szabo, PhD ^2, 5, 6^; Sondre Høeg Rukke, MS ^7^; Stener Nerland, MS ^1, 2^; Runar Elle Smelror, PhD ^1, 2^; Kjetil Nordbø Jørgensen, PhD ^2, 8^; Claudia Barth, PhD ^1, 2^; Dimitrios Andreou, MD PhD ^1, 2, 9^; Melissa A. Weibell, PhD ^10, 11^; Srdjan Djurovic, PhD ^3, 6, 12^; Ole A. Andreassen, MD PhD ^2, 5, 6^; Marianne Thoresen, MD PhD ^13, 14^; Gianluca Ursini, MD PhD ^15, 16^; Ingrid Agartz, MD PhD ^1, 2, 6, 9 •^ ; Stephanie Le Hellard, PhD ^3, 4 •^

^1^ Department of Psychiatric Research, Diakonhjemmet Hospital, Oslo, Norway

^2^ NORMENT, Institute of Clinical Medicine, University of Oslo, Oslo, Norway

^3^ NORMENT, Department of Clinical Science, University of Bergen, Bergen, Norway

^4^ Dr. Einar Martens Research Group for Biological Psychiatry, Center for Medical Genetics and Molecular Medicine, Haukeland University Hospital, Bergen, Norway

^5^ NORMENT, Division of Mental Health and Addiction, Oslo University Hospital, Oslo, Norway

^6^ KG Jebsen Centre for Neurodevelopmental Disorders, University of Oslo, Oslo, Norway

^7^ Faculty of Medicine, University of Bergen, Bergen, Norway

^8^ Department of Psychiatry, Telemark Hospital, Skien, Norway

^9^ Centre for Psychiatry Research, Department of Clinical Neuroscience, Karolinska Institutet and Stockholm Health Care Services, Stockholm County Council, Stockholm, Sweden

^10^ TIPS—Network for Clinical Research in Psychosis, Department of Psychiatry, Stavanger University Hospital, Stavanger, Norway,

^11^ Faculty of Health, Network for Medical Sciences, University of Stavanger, Stavanger, Norway

^12^ Department of Medical Genetics, Oslo University Hospital, Oslo, Norway

^13^ Department of Physiology, Institute of Basic Medical Sciences, University of Oslo, Oslo, Norway

^14^ Neonatal Neuroscience, Translational Health Sciences, University of Bristol, Bristol, United Kingdom

^15^ Lieber Institute for Brain Development, Johns Hopkins Medical Campus, Baltimore, MD

^16^ Department of Psychiatry and Behavioral Sciences, Johns Hopkins University School of Medicine, Baltimore, MD

* To whom correspondence should be addressed: Dr. Laura Anne Wortinger, Department of Psychiatric Research, Diakonhjemmet Hospital, Postbox 23 Vinderen, 0319, Oslo, Norway; e-mail: [l.a.w.bakke@medisin.uio.no](mailto:l.a.w.bakke@medisin.uio.no)

^#^ Equal contributions for first authorship

^•^ Equal contributions for last authorship

**Supplementary Figure 1:** Manhattan plot for differentially methylated positions (DMPs) associated with perinatal asphyxia.

**Supplementary Figure 2:** Scatterplot demonstrating the correlation between DNA methylation profiles associated with perinatal asphyxia in females (top) and males (bottom) between groups.

**Supplementary Figure 3-6:** DNA methylation of probes associated with *LINGO3, BLCAP;NNAT, NANOS2* and *SLC2A14* in whole blood significantly correlates with that in the brain at some genomic loci.

**Supplementary Figure 7:** Scatterplot demonstrating the correlation between DNA methylation profiles associated with perinatal asphyxia between patient subgroups.

**Supplementary Table 1:** Significant interaction between differentially methylated regions (DMRs) and perinatal asphyxia on Case/Control status in males

**Supplementary Table 2:** Significant interaction between differentially methylated regions (DMRs) and perinatal asphyxia on Case/Control status in females

**Supplementary Table 3:** Differentially methylated regions and perinatal asphyxia on disease severity

**Supplementary Table 4:** Differentially methylated regions and perinatal asphyxia on age of disease onset in males

**Supplementary Table 5:** Differentially methylated regions and perinatal asphyxia on Case/Control status in Schizophrenia

**Supplementary Table 6:** Differentially methylated regions and perinatal asphyxia on Case/Control status in Bipolar Disorder

**Supplementary Note 1:** Gestational age

**Supplementary Note 2:** Abbreviation List


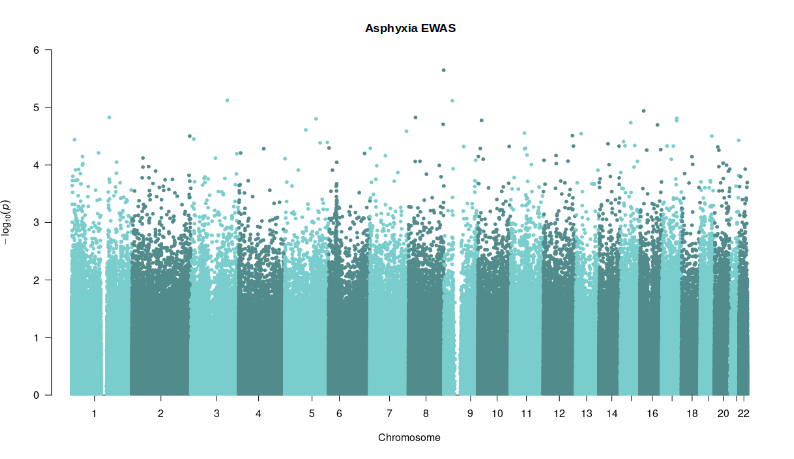


**Supplementary Figure 1:** Manhattan plot for differentially methylated positions (DMPs) associated with perinatal asphyxia.

**
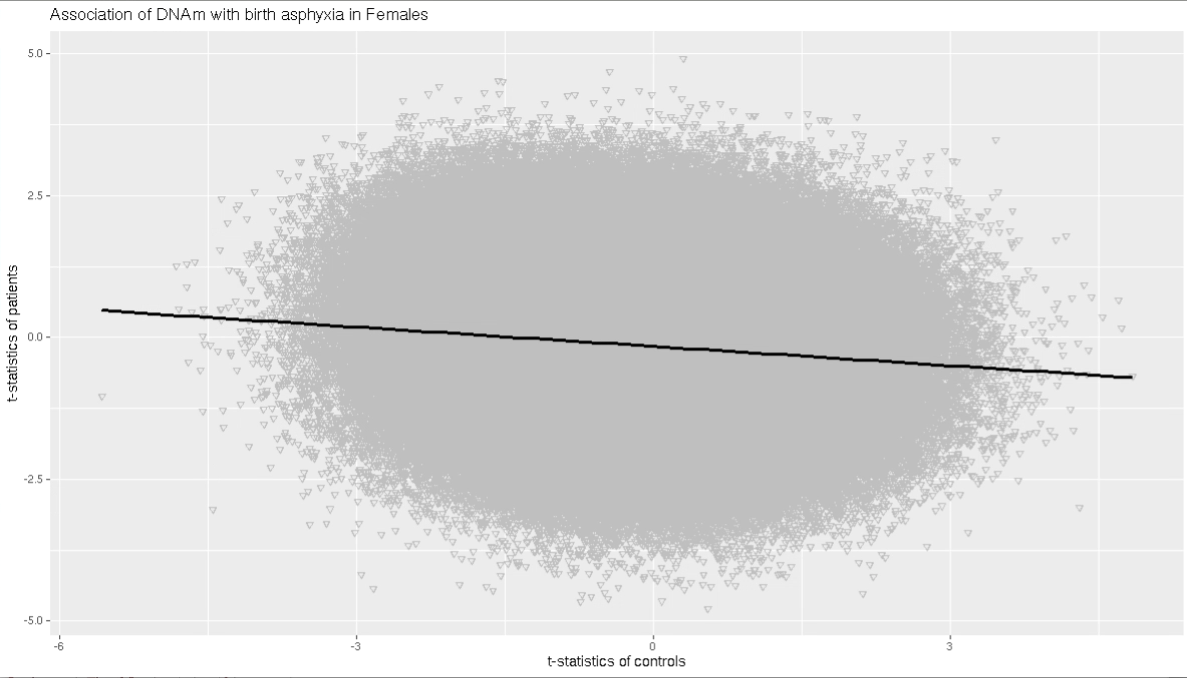
**

**
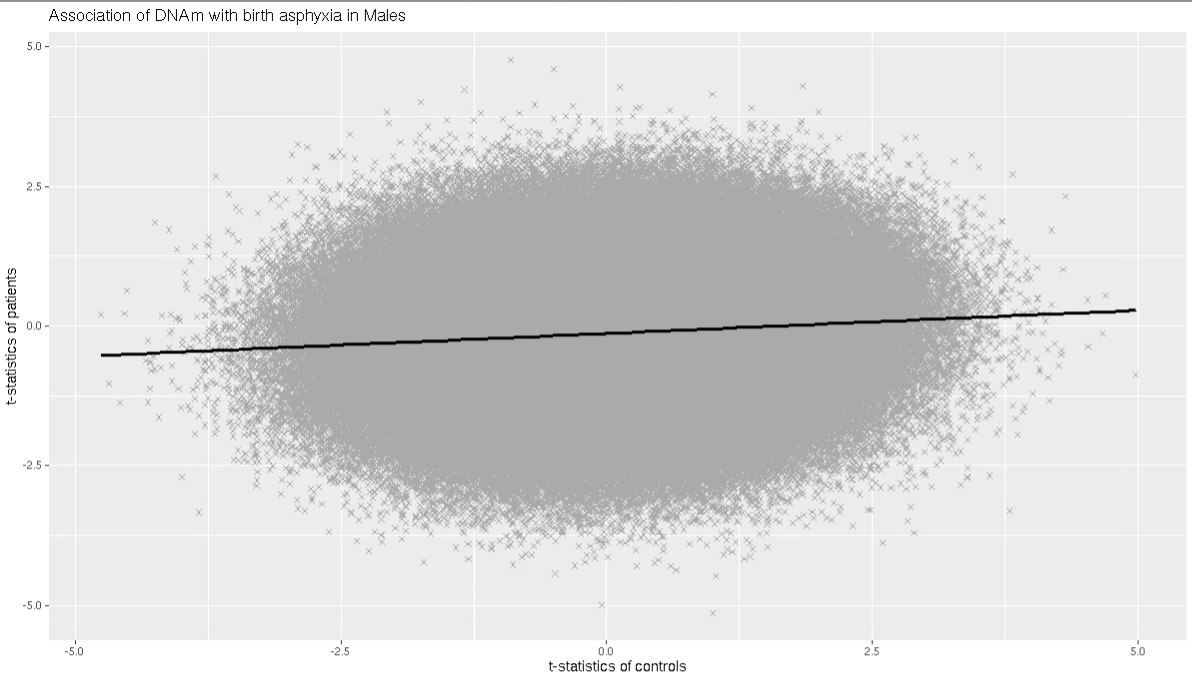
**

**Supplementary Figure 2:** Scatterplot demonstrating the correlation between DNA methylation profiles associated with perinatal asphyxia in females (top) and males (bottom) between groups.

**
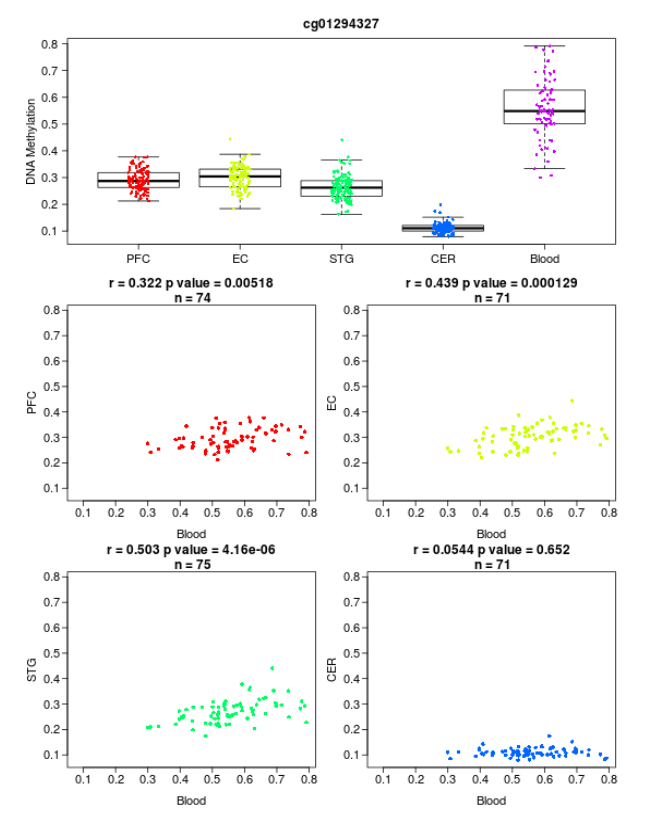
**

**Supplementary Figure 3:** DNA methylation correlation between blood and four different brain regions, retrieved from the searchable database by Hannon and colleagues (1). Shown is a boxplot of the distribution of DNA methylation values across all individuals split by tissue and four scatterplots demonstrating the relationship between DNA methylation of probe cg01294327 associated with *LINGO3* in whole blood and four brain regions (PFC, prefrontal cortex; EC, entorhinal cortex ; STG, superior temporal gyrus; CER, cerebellum). At this probe there is a significant correlation between individual variation in whole blood and that observed in the PFC, EC and STG.


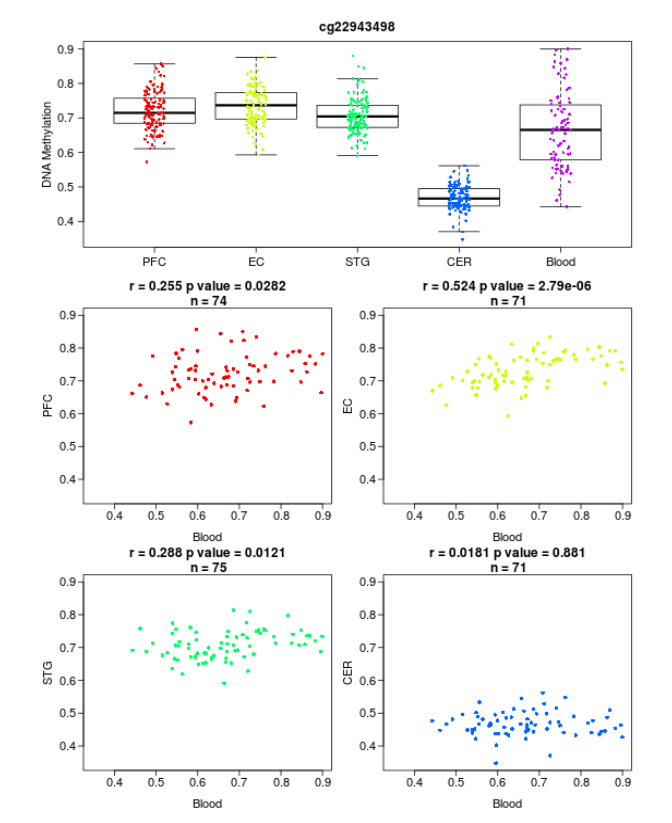


**Supplementary Figure 4:** DNA methylation correlation between blood and four different brain regions, retrieved from the searchable database by Hannon and colleagues (1). Shown is a boxplot of the distribution of DNA methylation values across all individuals split by tissue and four scatterplots demonstrating the relationship between DNA methylation of probe cg22943498 associated with *BLCAP;NNAT* in whole blood and four brain regions (PFC, prefrontal cortex; EC, entorhinal cortex ; STG, superior temporal gyrus; CER, cerebellum). At this probe there is a significant correlation between individual variation in whole blood and that observed in the PFC, EC and STG.

**
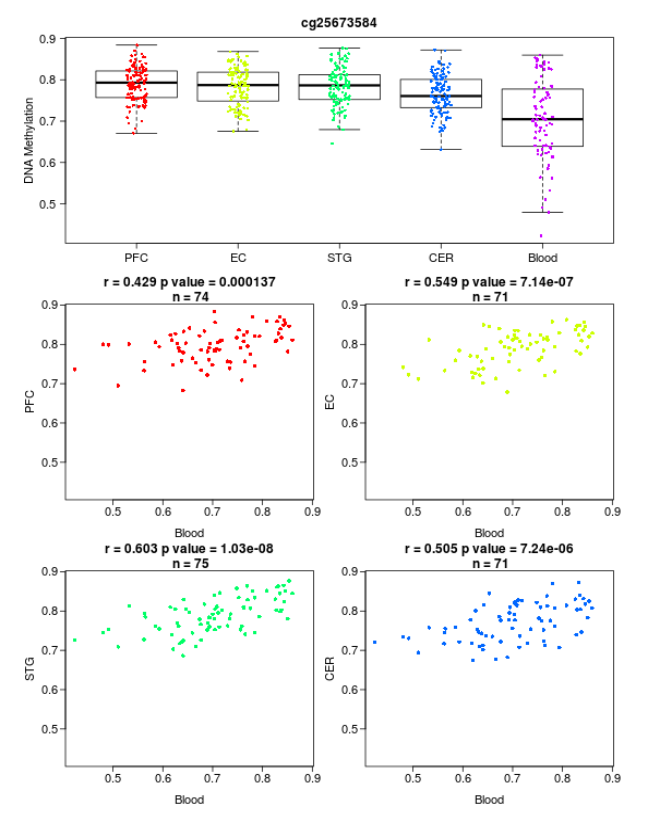
**

**Supplementary Figure 5:** DNA methylation correlation between blood and four different brain regions, retrieved from the searchable database by Hannon and colleagues (1). Shown is a boxplot of the distribution of DNA methylation values across all individuals split by tissue and four scatterplots demonstrating the relationship between DNA methylation of probe cg25673584 associated with *NANOS2* in whole blood and four brain regions (PFC, prefrontal cortex; EC, entorhinal cortex ; STG, superior temporal gyrus; CER, cerebellum). At this probe there is a significant correlation between individual variation in whole blood and that observed in all four brain regions.

**
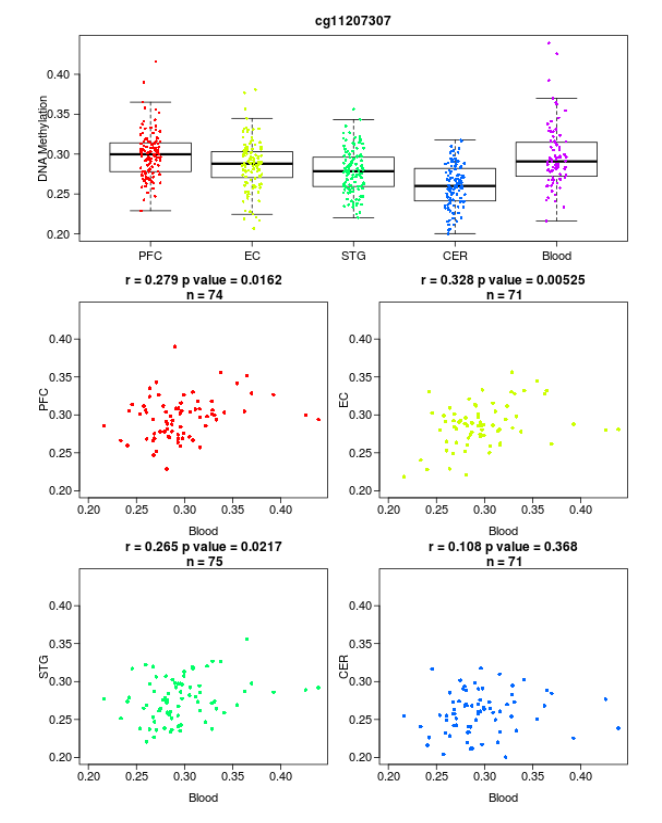
**

**Supplementary Figure 6:** DNA methylation correlation between blood and four different brain regions, retrieved from the searchable database by Hannon and colleagues (1). Shown is a boxplot of the distribution of DNA methylation values across all individuals split by tissue and four scatterplots demonstrating the relationship between DNA methylation of probe cg11207307 associated with *SLC2A14* in whole blood and four brain regions (PFC, prefrontal cortex; EC, entorhinal cortex ; STG, superior temporal gyrus; CER, cerebellum). At this probe there is a significant correlation between individual variation in whole blood and that observed in the PFC, EC and STG.

**
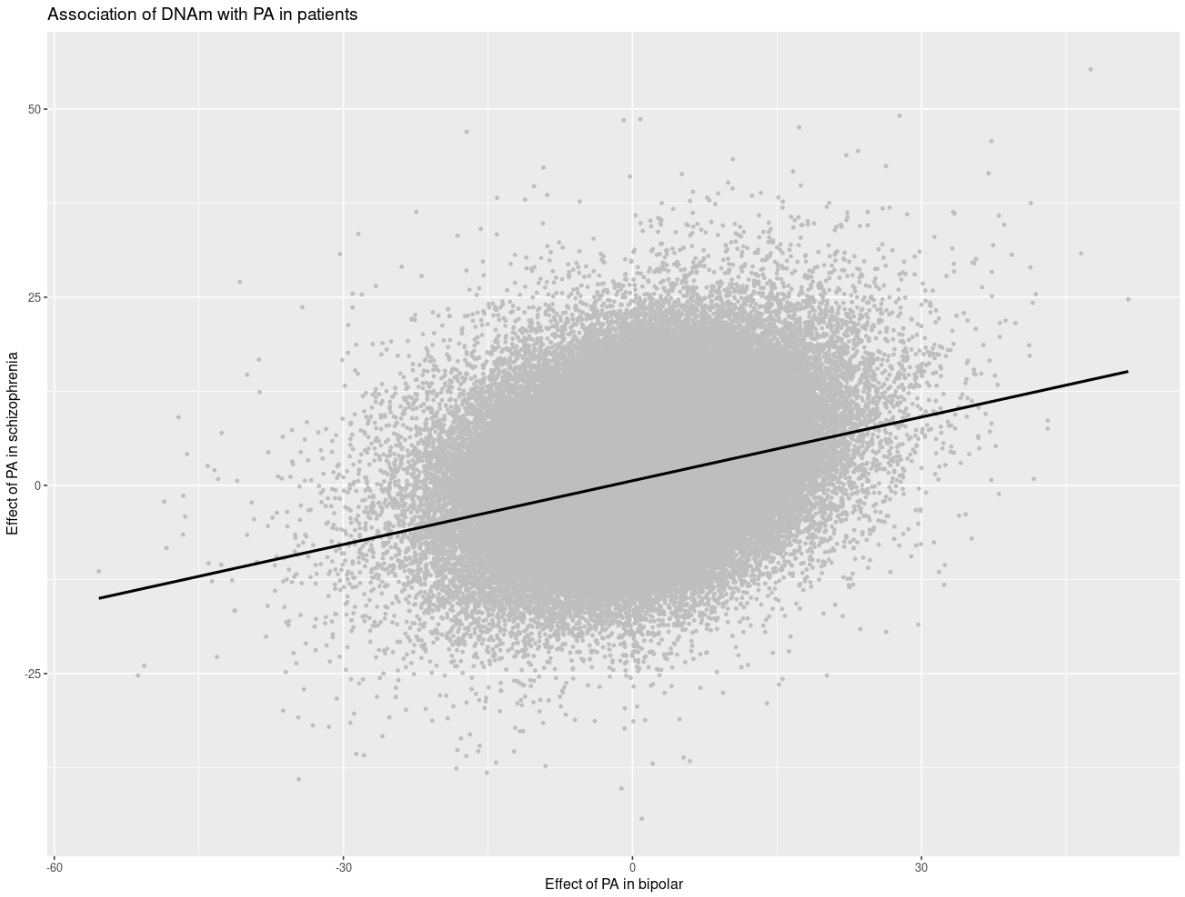
**

**Supplementary Figure 7:** Scatterplot demonstrating the correlation between DNA methylation profiles associated with perinatal asphyxia (PA) between patient subgroups.

**Supplementary Table 1:** Significant interaction between differentially methylated regions (DMRs) and perinatal asphyxia on Case/Control status in males

| Šidák *p* <.05 | | | | PA * DNAm | | | PA | | | DNAm | | |
| --- | --- | --- | --- | --- | --- | --- | --- | --- | --- | --- | --- | --- |
| chromosome | start | end | *gene* | Min *p* | n probes | Šidák *p* | Min *p* | n probes | Šidák *p* | Min *p* | n probes | Šidák *p* |
| 5 | 118603742 | 118603938 | *TNFAIP8* | 3.49E-05 | 4 | 1.78E-07 | 1.52E-05 | 4 | 7.75E-08 |  |  |  |
| 10 | 1704858 | 1705085 | *ADARB2* | 4.05E-03 | 3 | 2.01E-04 | 3.31E-02 | 3 | 2.33E-03 |  |  |  |
| 11 | 45825300 | 45825518 | *SLC35C1* | 4.05E-03 | 8 | 2.01E-04 |  |  |  |  |  |  |
| 16 | 50913874 | 50914024 |  | 4.05E-03 | 5 | 3.72E-04 |  |  |  |  |  |  |
| 4 | 186732837 | 186733060 | *SORBS2* | 1.46E-02 | 8 | 1.93E-03 |  |  |  |  |  |  |
| 12 | 2944286 | 2944493 | *NRIP2* | 1.46E-02 | 6 | 1.02E-02 |  |  |  |  |  |  |
| 3 | 39543544 | 39543776 | *MOBP* | 2.19E-02 | 5 | 3.17E-02 | 2.93E-03 | 6 | 2.61E-04 |  |  |  |
| 2 | 242802009 | 242802192 | *PDCD1* |  |  |  |  |  |  | 4.47E-07 | 5 | 1.22E-08 |
| 15 | 40268610 | 40268777 | *EIF2AK4* |  |  |  |  |  |  | 1.76E-06 | 4 | 8.43E-08 |
| 17 | 1665119 | 1665303 | *SERPINF1* |  |  |  |  |  |  | 7.25E-06 | 6 | 5.52E-07 |
| 17 | 75789279 | 75789529 |  |  |  |  |  |  |  | 2.89E-04 | 5 | 2.31E-05 |
| 6 | 166260319 | 166260572 |  |  |  |  |  |  |  | 8.83E-04 | 3 | 1.04E-04 |
| 12 | 131322960 | 131323042 | *STX2* |  |  |  |  |  |  | 5.18E-04 | 3 | 1.45E-04 |
| 20 | 5485144 | 5485511 | *LOC149837* |  |  |  |  |  |  | 2.72E-03 | 8 | 2.83E-04 |
| 17 | 75096202 | 75096382 |  |  |  |  |  |  |  | 2.71E-03 | 3 | 4.21E-04 |
| 2 | 239140032 | 239140369 | *LOC151174;LOC643387* |  |  |  |  |  |  | 3.65E-03 | 7 | 5.44E-04 |
| 15 | 69385753 | 69385944 | *EWSAT1;MIR548H4* |  |  |  |  |  |  | 2.92E-03 | 3 | 5.97E-04 |
| 16 | 50647607 | 50647798 | *NKD1* |  |  |  |  |  |  | 8.98E-03 | 3 | 2.57E-03 |
| 10 | 123355239 | 123355454 | *FGFR2* |  |  |  |  |  |  | 2.72E-03 | 3 | 2.83E-03 |
| 11 | 73116210 | 73116329 | *FAM168A* |  |  |  |  |  |  | 8.98E-03 | 3 | 4.29E-03 |
| 20 | 19915716 | 19915874 | *RIN2* |  |  |  |  |  |  | 5.12E-03 | 5 | 4.30E-03 |
| 16 | 85096433 | 85096666 | *KIAA0513* |  |  |  |  |  |  | 1.71E-02 | 4 | 9.55E-03 |
| 20 | 20248523 | 20248696 | *CFAP61* |  |  |  |  |  |  | 2.65E-02 | 3 | 1.34E-02 |
| 6 | 168393963 | 168394198 |  |  |  |  |  |  |  | 2.66E-02 | 4 | 1.62E-02 |
| 16 | 28518114 | 28518347 | *IL27* |  |  |  |  |  |  | 3.22E-02 | 6 | 1.62E-02 |
| 12 | 53443877 | 53443962 | *TNS2;LOC283335;TENC1* |  |  |  |  |  |  | 2.07E-02 | 3 | 1.93E-02 |
| 19 | 24097543 | 24097753 | *ZNF726* |  |  |  |  |  |  | 3.37E-02 | 4 | 1.97E-02 |
| 5 | 7519455 | 7519574 | *ADCY2* |  |  |  |  |  |  | 2.66E-02 | 3 | 2.10E-02 |
| 10 | 29194973 | 29195190 |  |  |  |  |  |  |  | 3.62E-02 | 3 | 2.15E-02 |
| 13 | 24825649 | 24825781 | *SPATA13* |  |  |  |  |  |  | 1.05E-02 | 3 | 3.01E-02 |
| 3 | 195943149 | 195943366 | *OSTalpha;SLC51A* |  |  |  |  |  |  | 2.65E-02 | 5 | 3.24E-02 |
| 17 | 1686627 | 1686737 | *SMYD4* |  |  |  |  |  |  | 3.22E-02 | 3 | 3.26E-02 |
| 14 | 65204509 | 65204625 | *PLEKHG3* |  |  |  |  |  |  | 3.23E-02 | 3 | 3.34E-02 |
| 1 | 40025232 | 40025415 | *LOC728448* |  |  |  |  |  |  | 4.76E-02 | 3 | 3.82E-02 |
| 2 | 220264753 | 220264849 |  |  |  |  |  |  |  | 3.40E-02 | 3 | 4.39E-02 |

Positions of DMRs are given according to hg19 reference genome with chromosome: start-end. DMRs were identified using a seed with a *p* value of < 0.05. For each DMR, the table lists the minimum *p* value (Min *p*), the number of probes (n probes) and the Šidák corrected *p* value (Šidák *p*). PA (perinatal asphyxia)

**Supplementary Table 2:** Significant interaction between differentially methylated regions (DMRs) and perinatal asphyxia on Case/Control status in females

| Šidák *p* <.05 | | | | PA * DNAm | | | DNAm | | |
| --- | --- | --- | --- | --- | --- | --- | --- | --- | --- |
| chromosome | start | end | *gene* | Min *p* | n probes | Šidák *p* | Min *p* | n probes | Šidák *p* |
| 2 | 183943319 | 183943698 | *DUSP19* | 8.31E-03 | 7 | 9.31E-04 |  |  |  |
| 1 | 35586358 | 35586588 |  | 2.70E-02 | 4 | 1.87E-03 |  |  |  |
| 3 | 195538675 | 195538961 | *MUC4* |  |  |  | 3.40E-05 | 6 | 9.97E-07 |
| 8 | 23563859 | 23564193 | *NKX2-6* |  |  |  | 4.78E-05 | 8 | 6.03E-06 |
| 16 | 1480790 | 1480970 | *C16orf91* |  |  |  | 2.24E-04 | 4 | 2.07E-05 |
| 1 | 42384284 | 42384647 | *HIVEP3* |  |  |  | 3.63E-04 | 8 | 3.73E-05 |
| 14 | 58905592 | 58905740 | *KIAA0586* |  |  |  | 4.05E-04 | 3 | 9.21E-05 |
| 17 | 39969239 | 39969397 | *SC65;FKBP10;P3H4* |  |  |  | 8.14E-04 | 6 | 2.27E-04 |
| 2 | 220436856 | 220436948 | *OBSL1;INHA* |  |  |  | 3.40E-05 | 5 | 2.56E-04 |
| 1 | 223566643 | 223566794 | *C1orf65;CCDC185* |  |  |  | 4.05E-04 | 6 | 2.71E-04 |
| 6 | 28226885 | 28227220 | *NKAPL;ZKSCAN4* |  |  |  | 2.24E-04 | 11 | 2.84E-04 |
| 20 | 39766765 | 39766794 | *PLCG1-AS1;PLCG1* |  |  |  | 3.63E-04 | 3 | 3.14E-04 |
| 2 | 204801413 | 204801510 | *ICOS* |  |  |  | 1.30E-03 | 4 | 6.83E-04 |
| 20 | 35233380 | 35233582 | *C20orf24;TGIF2-C20orf24* |  |  |  | 2.36E-03 | 4 | 7.12E-04 |
| 20 | 35422495 | 35422703 | *C20orf117* |  |  |  | 6.96E-03 | 3 | 2.44E-03 |
| 1 | 55271673 | 55271927 | *C1orf177* |  |  |  | 7.78E-03 | 7 | 2.59E-03 |
| 19 | 37825211 | 37825572 | *HKR1* |  |  |  | 4.38E-03 | 9 | 3.13E-03 |
| 8 | 143859669 | 143859906 | *LYNX1* |  |  |  | 8.07E-03 | 7 | 3.49E-03 |
| 17 | 79817079 | 79817271 | *P4HB* |  |  |  | 8.64E-03 | 4 | 4.29E-03 |
| 2 | 210444075 | 210444364 | *MAP2* |  |  |  | 9.49E-03 | 7 | 4.92E-03 |
| 3 | 3152201 | 3152530 | *IL5RA* |  |  |  | 2.91E-03 | 3 | 5.30E-03 |
| 9 | 117266783 | 117266918 | *DFNB31* |  |  |  | 1.87E-02 | 3 | 2.19E-02 |
| 18 | 13611190 | 13611576 | *LDLRAD4;C18orf1;MIR4526* |  |  |  | 2.16E-02 | 8 | 2.41E-02 |
| 10 | 119184448 | 119184664 |  |  |  |  | 3.07E-02 | 4 | 3.03E-02 |
| 1 | 2949633 | 2949673 |  |  |  |  | 1.20E-02 | 3 | 3.67E-02 |
| 7 | 27183591 | 27183950 | *HOXA5* |  |  |  | 1.23E-02 | 7 | 4.19E-02 |
| 4 | 107236270 | 107236485 | *AIMP1;TBCK* |  |  |  | 4.03E-02 | 3 | 4.38E-02 |

Positions of DMRs are given according to hg19 reference genome with chromosome: start-end. DMRs were identified using a seed with a *p* value of < 0.05. For each DMR, the table lists the minimum *p* value (Min *p*), the number of probes (n probes) and the Šidák corrected *p* value (Šidák *p*). PA (perinatal asphyxia)

**Supplementary Table 3:** Differentially methylated regions and perinatal asphyxia on disease severity

|  | Šidák *p* <.05 | | | | PA * DNAm | | | DNAm | | |
| --- | --- | --- | --- | --- | --- | --- | --- | --- | --- | --- |
|  | chromosome | start | end | *gene* | Min *p* | n probes | Šidák *p* | Min *p* | n probes | Šidák *p* |
| males |  |  |  |  |  |  |  |  |  |  |
|  | 1 | 59042971 | 59043280 | *TACSTD2* | 1.53E-04 | 7 | 3.45E-05 |  |  |  |
|  | 6 | 164506789 | 164507305 |  |  |  |  | 6.59E-04 | 8 | 5.88E-08 |
|  | 13 | 113242878 | 113243279 | *TUBGCP3* |  |  |  | 1.62E-04 | 6 | 1.05E-06 |
|  | 6 | 29648161 | 29648628 |  |  |  |  | 2.72E-04 | 17 | 5.60E-06 |
|  | 19 | 40919245 | 40919485 | *PRX* |  |  |  | 1.57E-03 | 3 | 1.31E-04 |
|  | 2 | 27301252 | 27301597 | *EMILIN1* |  |  |  | 1.08E-03 | 5 | 1.53E-04 |
|  | 1 | 200842126 | 200842282 | *GPR25* |  |  |  | 1.68E-03 | 3 | 2.40E-04 |
|  | 12 | 131519883 | 131520095 | *ADGRD1;GPR133* |  |  |  | 2.18E-03 | 5 | 3.29E-04 |
|  | 12 | 131401275 | 131401509 |  |  |  |  | 2.97E-03 | 3 | 7.95E-04 |
|  | 6 | 31939025 | 31939322 | *STK19;DOM3Z* |  |  |  | 1.37E-03 | 10 | 1.02E-03 |
|  | 17 | 1478463 | 1478604 | *SLC43A2* |  |  |  | 4.05E-03 | 3 | 1.21E-03 |
|  | 7 | 44795713 | 44795780 | *ZMIZ2* |  |  |  | 2.50E-03 | 3 | 6.31E-03 |
|  | 17 | 2294951 | 2295150 | *MNT* |  |  |  | 2.21E-02 | 3 | 9.82E-03 |
|  | 16 | 29832601 | 29832769 | *PAGR1;MVP* |  |  |  | 2.57E-02 | 4 | 1.51E-02 |
|  | 19 | 12958803 | 12958913 | *MAST1* |  |  |  | 2.18E-02 | 4 | 1.69E-02 |
|  | 6 | 32014381 | 32014605 | *TNXB* |  |  |  | 6.55E-03 | 4 | 1.81E-02 |
|  | 21 | 40759534 | 40759694 | *WRB* |  |  |  | 2.81E-02 | 6 | 1.85E-02 |
|  | 3 | 196705742 | 196705898 |  |  |  |  | 1.06E-02 | 5 | 1.95E-02 |
|  | 11 | 47282968 | 47283025 | *NR1H3* |  |  |  | 2.05E-02 | 3 | 2.80E-02 |
|  | 8 | 17433694 | 17433926 | *PDGFRL* |  |  |  | 2.85E-02 | 6 | 3.22E-02 |
|  | 11 | 1475775 | 1475899 | *BRSK2* |  |  |  | 2.81E-02 | 3 | 4.66E-02 |
| females |  |  |  |  |  |  |  |  |  |  |
|  | 1 | 236686618 | 236686849 | *LGALS8;LGALS8-AS1* | 5.64E-03 | 8 | 2.27E-04 |  |  |  |
|  | 7 | 27183133 | 27184316 | *HOXA5;HOXA-AS3* |  |  |  | 1.45E-05 | 24 | 1.09E-10 |
|  | 17 | 6796745 | 6797708 | *ALOX12P2* |  |  |  | 7.67E-04 | 8 | 4.00E-09 |
|  | 16 | 2907695 | 2908245 | *PRSS22* |  |  |  | 3.02E-05 | 10 | 1.14E-08 |
|  | 19 | 38794514 | 38794845 | *YIF1B;C19orf33* |  |  |  | 1.45E-05 | 7 | 2.09E-07 |
|  | 22 | 42095347 | 42095536 | *MEI1* |  |  |  | 2.21E-05 | 6 | 1.59E-06 |
|  | 4 | 184961220 | 184961374 |  |  |  |  | 2.51E-05 | 3 | 1.95E-06 |
|  | 6 | 31690998 | 31691442 | *C6orf25;LY6G6C* |  |  |  | 2.57E-04 | 9 | 1.68E-05 |
|  | 6 | 33091407 | 33091841 | *HLA-DPB2* |  |  |  | 7.67E-04 | 7 | 6.49E-05 |
|  | 1 | 159770136 | 159770368 | *FCRL6* |  |  |  | 8.57E-04 | 5 | 1.37E-04 |
|  | 11 | 75921940 | 75922323 |  |  |  |  | 1.17E-03 | 5 | 1.72E-04 |
|  |  |  |  |  |  |  |  |  |  |  |
|  | 1 | 95698827 | 95699097 | *RWDD3* |  |  |  | 3.07E-03 | 6 | 7.59E-04 |
|  | 20 | 5485144 | 5485294 | *LOC149837* |  |  |  | 2.19E-03 | 6 | 7.60E-04 |
|  | 5 | 140787623 | 140787864 | *PCDHGA4* |  |  |  | 3.99E-03 | 3 | 1.22E-03 |
|  | 11 | 102638432 | 102638706 |  |  |  |  | 3.99E-03 | 5 | 1.34E-03 |
|  | 19 | 695447 | 695642 | *PRSS57;PRSSL1* |  |  |  | 3.99E-03 | 6 | 1.65E-03 |
|  | 1 | 120438925 | 120439138 | *ADAM30* |  |  |  | 2.33E-03 | 6 | 2.03E-03 |
|  | 1 | 1148973 | 1149192 | *TNFRSF4* |  |  |  | 7.97E-03 | 3 | 4.39E-03 |
|  | 2 | 198651076 | 198651356 | *BOLL* |  |  |  | 1.85E-03 | 6 | 5.21E-03 |
|  | 10 | 5638081 | 5638276 |  |  |  |  | 9.95E-03 | 3 | 6.56E-03 |
|  | 1 | 202172778 | 202172912 | *LGR6* |  |  |  | 7.97E-03 | 5 | 7.29E-03 |
|  | 8 | 124191131 | 124191241 | *FAM83A* |  |  |  | 7.74E-03 | 3 | 7.65E-03 |
|  | 7 | 44888622 | 44888679 | *H2AFV* |  |  |  | 4.78E-03 | 3 | 8.10E-03 |
|  | 1 | 236686618 | 236686849 | *LGALS8;LGALS8-AS1* |  |  |  | 9.78E-03 | 8 | 8.15E-03 |
|  | 21 | 45705543 | 45705742 | *AIRE* |  |  |  | 3.99E-03 | 8 | 8.29E-03 |
|  | 13 | 112627458 | 112627641 |  |  |  |  | 1.37E-02 | 3 | 1.15E-02 |
|  | 11 | 131630464 | 131630648 | *NTM* |  |  |  | 1.77E-02 | 3 | 1.70E-02 |
|  | 1 | 68512777 | 68512971 | *DIRAS3* |  |  |  | 9.51E-04 | 5 | 1.74E-02 |
|  | 16 | 3062296 | 3062597 | *CLDN9* |  |  |  | 1.38E-02 | 6 | 2.12E-02 |
|  | 4 | 186732837 | 186733060 | *SORBS2* |  |  |  | 3.46E-02 | 8 | 3.62E-02 |
|  | 19 | 35490470 | 35490624 | *GRAMD1A* |  |  |  | 2.87E-02 | 4 | 3.92E-02 |
|  | 16 | 69966815 | 69967063 | *MIR140;WWP2* |  |  |  | 1.88E-02 | 5 | 4.25E-02 |
|  | 12 | 2944286 | 2944493 | *NRIP2* |  |  |  | 2.53E-02 | 6 | 4.30E-02 |

Positions of DMRs are given according to hg19 reference genome with chromosome: start-end. DMRs were identified using a seed with a *p* value of < 0.05. For each DMR, the table lists the minimum *p* value (Min *p*), the number of probes (n probes) and the Šidák corrected *p* value (Šidák *p*). PA (perinatal asphyxia)

**Supplementary Table 4:** Differentially methylated regions and perinatal asphyxia on age of disease onset in males

| Šidák p <.05 | | | | PA * DNAm | | | PA | | | DNAm | | |
| --- | --- | --- | --- | --- | --- | --- | --- | --- | --- | --- | --- | --- |
| chromosome | start | end | *gene* | Min *p* | n probes | Šidák *p* | Min *p* | n probes | Šidák *p* | Min *p* | n probes | Šidák *p* |
| 5 | 110062384 | 110062837 | *TMEM232* | 2.08E-06 | 10 | 3.57E-08 | 2.33E-04 | 5 | 2.45E-03 |  |  |  |
| 17 | 3289363 | 3289798 |  |  |  |  |  |  |  | 1.25E-05 | 6 | 1.53E-07 |
| 2 | 206628415 | 206628773 | *NRP2* |  |  |  |  |  |  | 9.48E-06 | 9 | 1.86E-07 |
| 1 | 59042971 | 59043370 | *TACSTD2* |  |  |  |  |  |  | 1.25E-05 | 8 | 7.02E-07 |
| 22 | 50585161 | 50585538 | *MOV10L1* |  |  |  |  |  |  | 7.96E-07 | 10 | 7.36E-07 |
| 19 | 11784514 | 11784955 | *ZNF833* |  |  |  |  |  |  | 2.14E-05 | 8 | 1.00E-06 |
| 2 | 198651111 | 198651590 | *BOLL* |  |  |  |  |  |  | 4.14E-05 | 8 | 3.68E-06 |
| 1 | 153599479 | 153600064 | *S100A13;S100A1* |  |  |  |  |  |  | 2.30E-04 | 13 | 6.68E-06 |
| 17 | 2169571 | 2169989 | *SMG6* |  |  |  |  |  |  | 1.64E-03 | 5 | 1.19E-05 |
| 8 | 144120335 | 144120681 | *C8orf31* |  |  |  |  |  |  | 2.08E-04 | 7 | 3.39E-05 |
| 5 | 131808798 | 131808970 | *C5orf56* |  |  |  |  |  |  | 2.08E-04 | 3 | 4.92E-05 |
| 1 | 26233332 | 26233709 | *STMN1* |  |  |  |  |  |  | 1.17E-03 | 10 | 2.10E-04 |
| 9 | 140197553 | 140197890 | *NRARP* |  |  |  |  |  |  | 1.48E-03 | 4 | 3.10E-04 |
| 1 | 38022316 | 38022607 | *DNALI1* |  |  |  |  |  |  | 1.63E-03 | 9 | 4.50E-04 |
| 3 | 39543515 | 39543776 | *MOBP* |  |  |  |  |  |  | 2.30E-03 | 6 | 8.75E-04 |
| 16 | 86229698 | 86229910 |  |  |  |  |  |  |  | 3.58E-03 | 3 | 1.54E-03 |
| 6 | 41068553 | 41068741 | *NFYA;LOC221442* |  |  |  |  |  |  | 5.34E-03 | 4 | 2.93E-03 |
| 19 | 8591721 | 8591776 | *MYO1F* |  |  |  |  |  |  | 2.26E-03 | 2 | 3.11E-03 |
| 12 | 1025529 | 1025772 | *RAD52* |  |  |  |  |  |  | 6.73E-03 | 4 | 3.21E-03 |
| 22 | 32058500 | 32058810 |  |  |  |  |  |  |  | 7.27E-03 | 6 | 3.43E-03 |
| 20 | 11899739 | 11899859 | *BTBD3* |  |  |  |  |  |  | 5.07E-03 | 3 | 4.82E-03 |
| 22 | 45809793 | 45810043 | *RIBC2;SMC1B* |  |  |  |  |  |  | 1.11E-03 | 3 | 5.17E-03 |
| 11 | 86142407 | 86142587 |  |  |  |  |  |  |  | 8.14E-03 | 4 | 5.82E-03 |
| 22 | 50528213 | 50528312 | *MOV10L1* |  |  |  |  |  |  | 1.41E-03 | 4 | 1.02E-02 |
| 13 | 21900392 | 21900591 |  |  |  |  |  |  |  | 4.67E-03 | 4 | 1.41E-02 |
| 16 | 87781336 | 87781501 | *KLHDC4* |  |  |  |  |  |  | 1.65E-02 | 4 | 1.50E-02 |
| 1 | 228291486 | 228291705 | *C1orf35* |  |  |  |  |  |  | 2.13E-02 | 4 | 1.69E-02 |
| 20 | 25129457 | 25129562 | *LOC284798* |  |  |  |  |  |  | 4.07E-03 | 6 | 3.27E-02 |
| 6 | 32116841 | 32117088 | *PRRT1* |  |  |  |  |  |  | 3.34E-02 | 10 | 3.65E-02 |
| 16 | 8806531 | 8806756 | *ABAT* |  |  |  |  |  |  | 5.34E-03 | 8 | 3.87E-02 |
| 12 | 46660255 | 46660469 | *SLC38A1* |  |  |  |  |  |  | 4.16E-02 | 4 | 4.39E-02 |

Positions of DMRs are given according to hg19 reference genome with chromosome: start-end. DMRs were identified using a seed with a *p* value of < 0.05. For each DMR, the table lists the minimum *p* value (Min *p*), the number of probes (n probes) and the Šidák corrected *p* value (Šidák *p*). PA (perinatal asphyxia)

**Supplementary Table 5:** Differentially methylated regions and perinatal asphyxia on Case/Control status in Schizophrenia

| Šidák p <.05 | | | | PA * DNAm | | | PA | | | DNAm | | |
| --- | --- | --- | --- | --- | --- | --- | --- | --- | --- | --- | --- | --- |
| chromosome | start | end | gene | Min p | n probes | Šidák p | Min p | n probes | Šidák p | Min p | n probes | Šidák p |
| 7 | 27170313 | 27171051 | *HOXA4* | 1.69E-03 | 13 | 2.70E-05 | 5.47E-04 | 9 | 6.05E-07 |  |  |  |
| 12 | 8025394 | 8025646 | *SLC2A14* | 1.69E-03 | 4 | 6.34E-02 | 6.87E-03 | 4 | 1.98E-03 |  |  |  |
| 4 | 1243849 | 1244086 | *CTBP1;C4orf42* | 3.52E-03 | 6 | 2.08E-04 |  |  |  |  |  |  |
| 3 | 39543515 | 39543776 | *MOBP* | 6.67E-03 | 6 | 1.31E-03 | 1.22E-02 | 6 | 4.15E-04 |  |  |  |
| 17 | 79380493 | 79380706 | *BAHCC1* | 9.85E-03 | 4 | 1.79E-03 |  |  |  |  |  |  |
| 10 | 4230567 | 4230752 |  | 2.49E-03 | 4 | 3.91E-03 |  |  |  |  |  |  |
| 19 | 46417551 | 46417734 | *NANOS2* | 4.86E-02 | 4 | 1.29E-02 |  |  |  |  |  |  |

Positions of DMRs are given according to hg19 reference genome with chromosome: start-end. DMRs were identified using a seed with a *p* value of < 0.05. For each DMR, the table lists the minimum *p* value (Min *p*), the number of probes (n probes) and the Šidák corrected *p* value (Šidák *p*). PA (perinatal asphyxia)

**Supplementary Table 6:** Differentially methylated regions and perinatal asphyxia on Case/Control status in Bipolar Disorder

| Šidák p <.05 | | | | PA * DNAm | | | PA | | | DNAm | | |
| --- | --- | --- | --- | --- | --- | --- | --- | --- | --- | --- | --- | --- |
| chromosome | start | end | gene | Min p | n probes | Šidák p | Min p | n probes | Šidák p | Min p | n probes | Šidák p |
| 19 | 37825211 | 37825455 | *HKR1* | 1.11E-02 | 5 | 5.09E-03 | 3.25E-04 | 9 | 3.08E-02 | 9.90E-04 | 9 | 9.78E-04 |
| 3 | 169530692 | 169530875 | *LRRC34* | 1.11E-02 | 3 | 4.26E-02 |  |  |  |  |  |  |
| 3 | 113955940 | 113955994 | *ZNF80* |  |  |  | 2.20E-02 | 2 | 7.20E-03 |  |  |  |
| 20 | 36148738 | 36149022 | *BLCAP;NNAT* |  |  |  | 2.20E-02 | 13 | 3.62E-02 |  |  |  |

Positions of DMRs are given according to hg19 reference genome with chromosome: start-end. DMRs were identified using a seed with a *p* value of < 0.05. For each DMR, the table lists the minimum *p* value (Min *p*), the number of probes (n probes) and the Šidák corrected *p* value (Šidák *p*). PA (perinatal asphyxia)

**Supplementary Note 1:** Gestational age

We cross-referenced the CpGs within the DMRs that we report to be associated with asphyxia together with CpGs reported to be associated with GA in studies from the EWAS catalog (2-5). 64040 CpGs overlapped with GA. We found that two of the three probes in the DMR annotated to *LINGO3*, where we found an interaction between DNAm and asphyxia on the case control status, have also been shown to be associated with GA. The same is true for 7 out of 8 probes in the DMR annotated to *SLC35C1*, where we found an interaction between DNAm and asphyxia on the case control status in males. In addition, 1 of the 10 CpGs in the DMR annotated to *TMEM232*, 1 of the 8 CpGs in the DMR annotated to *SORBS2*, 1 of the 6 probes in the DMR annotated to *NRIP2* have also been found to be associated with GA.

**Supplementary Note 2:** Abbreviation List

BD bipolar disorders

CpGs cytosine-phosphate-guanine sites

DMPs differentially methylated positions

DNAm DNA methylation

EWAS epigenome wide association study

GWAS genome-wide association studies

MBRN Medical Birth Registry of Norway

PA Perinatal asphyxia

PANSS Positive and Negative Syndrome Scale

PCs principal components

SZ schizophrenia

SNPs single nucleotide polymorphisms

TIPS Early Diagnostic and Treatment of Psychosis

TOP Thematically Organized Psychosis

Youth-TOP Youth Thematic Organized Psychosis

**References**

1. Hannon E, Lunnon K, Schalkwyk L, Mill J. Interindividual methylomic variation across blood, cortex, and cerebellum: implications for epigenetic studies of neurological and neuropsychiatric phenotypes. Epigenetics. 2015;10:1024-1032.

2. Bohlin J, Haberg SE, Magnus P, Reese SE, Gjessing HK, Magnus MC, Parr CL, Page CM, London SJ, Nystad W. Prediction of gestational age based on genome-wide differentially methylated regions. Genome Biol. 2016;17:207.

3. Hannon E, Schendel D, Ladd-Acosta C, Grove J, Hansen CS, Hougaard DM, Bresnahan M, Mors O, Hollegaard MV, Baekvad-Hansen M, Hornig M, Mortensen PB, Borglum AD, Werge T, Pedersen MG, Nordentoft M, i P-BASDG, Buxbaum JD, Daniele Fallin M, Bybjerg-Grauholm J, Reichenberg A, Mill J. Variable DNA methylation in neonates mediates the association between prenatal smoking and birth weight. Philos Trans R Soc Lond B Biol Sci. 2019;374:20180120.

4. Kashima K, Kawai T, Nishimura R, Shiwa Y, Urayama KY, Kamura H, Takeda K, Aoto S, Ito A, Matsubara K, Nagamatsu T, Fujii T, Omori I, Shimizu M, Hyodo H, Kugu K, Matsumoto K, Shimizu A, Oka A, Mizuguchi M, Nakabayashi K, Hata K, Takahashi N. Identification of epigenetic memory candidates associated with gestational age at birth through analysis of methylome and transcriptional data. Sci Rep. 2021;11:3381.

5. Spiers H, Hannon E, Schalkwyk LC, Smith R, Wong CC, O'Donovan MC, Bray NJ, Mill J. Methylomic trajectories across human fetal brain development. Genome Res. 2015;25:338-352.
